# Beyond AOPs: A Mechanistic Evaluation of NAMs in DART Testing

**DOI:** 10.3389/ftox.2022.838466

**Published:** 2022-03-07

**Authors:** Ramya Rajagopal, Maria T. Baltazar, Paul L. Carmichael, Matthew P. Dent, Julia Head, Hequn Li, Iris Muller, Joe Reynolds, Kritika Sadh, Wendy Simpson, Sandrine Spriggs, Andrew White, Predrag Kukic

**Affiliations:** Unilever Safety and Environmental Assurance Centre, Colworth Science Park, Sharnbrook, United Kingdom

**Keywords:** DART, NAMs, non-animal alternatives, NGRA, mechanistic evaluation

## Abstract

New Approach Methodologies (NAMs) promise to offer a unique opportunity to enable human-relevant safety decisions to be made without the need for animal testing in the context of exposure-driven Next Generation Risk Assessment (NGRA). Protecting human health against the potential effects a chemical may have on embryo-foetal development and/or aspects of reproductive biology using NGRA is particularly challenging. These are not single endpoint or health effects and risk assessments have traditionally relied on data from Developmental and Reproductive Toxicity (DART) tests in animals. There are numerous Adverse Outcome Pathways (AOPs) that can lead to DART, which means defining and developing strict testing strategies for every AOP, to predict apical outcomes, is neither a tenable goal nor a necessity to ensure NAM-based safety assessments are fit-for-purpose. Instead, a pragmatic approach is needed that uses the available knowledge and data to ensure NAM-based exposure-led safety assessments are sufficiently protective. To this end, the mechanistic and biological coverage of existing NAMs for DART were assessed and gaps to be addressed were identified, allowing the development of an approach that relies on generating data relevant to the overall mechanisms involved in human reproduction and embryo-foetal development. Using the knowledge of cellular processes and signalling pathways underlying the key stages in reproduction and development, we have developed a broad outline of endpoints informative of DART. When the existing NAMs were compared against this outline to determine whether they provide comprehensive coverage when integrated in a framework, we found them to generally cover the reproductive and developmental processes underlying the traditionally evaluated apical endpoint studies. The application of this safety assessment framework is illustrated using an exposure-led case study.

## 1 Introduction

The value of using New Approach Methodologies (NAMs) as alternatives to animal testing in the evaluation of chemical safety has gained much attention and recognition (ECC/HC, 2016; ECHA, 2016; ECHA, 2017; EPA, 2018). To foster their development and application, various agencies have put forward guidance, frameworks and workplans that ensure confidence, consistency and are fit-for-purpose, when generating NAMs hazard data for various purposes (OECD, 2005; OECD, 2017; OECD, 2018; Parish et al., 2020). The International Cooperation on Cosmetics Regulation (ICCR) has outlined key principles that guide risk assessors to use NAMs in an integrated manner for Next Generation Risk Assessment (NGRA) (Dent et al., 2018). There is also additional guidance published on specific NAMs that can be combined in a risk assessment of cosmetic ingredients, in a tiered manner, aligned to the different tiers of the SEURAT-1 *ab initio* workflow for systemic repeat-dose toxicity (Berggren et al., 2017; ICCR, 2018). Case studies are being generated to illustrate the practical application of these principles in assessing the safety of cosmetic products and demonstrating that NAMs can provide valuable insights into non-animal safety assessment (Baltazar et al., 2020).

For many substances, data covering developmental and reproductive toxicity (DART) are lacking, and filling these gaps using traditional methods would use a vast number of experimental animals (Rovida and Hartung, 2009). The development of NAMs to address DART effects is therefore a high priority. Several projects have evaluated the predictive value of batteries of alternative methods for DART, that target a select set of mechanisms and adverse outcome pathways (AOPs). The ReProTect project, which was part of the 6th European Framework Program, published the findings of its feasibility study where 10 chemicals were tested in a set of 14 assays. Based on a nearest neighbour and weight of evidence approach, the results were used to predict any adverse effects on fertility and embryonic development (Schenk et al., 2010). The 7th European Framework Program’s ChemScreen project published the DART outcome of testing 12 compounds on a set of 31 assays. Toxicokinetic modelling was included to validate the *in vitro* to *in vivo* dose comparisons and an *in silico* pre-screening module was also applied to reduce testing needs (Piersma et al., 2013; van der Burg et al., 2015). These important studies shared a common goal; to use NAMs to predict whether a substance will be a reproductive or developmental toxicant *in vivo*, often using animal test data as a benchmark. However, in many cases the uptake of these methods into regulatory decision making has been slow, which is understandable given the complexities of trying to use individual NAMs to predict apical endpoints in an intact organism. In recent years, a lot of emphasis has been placed on using AOPs to organise the mechanistic data (including data generated using NAMs) to facilitate safety decision making and either predict or explain adverse outcomes (Ankley et al., 2010; Meek et al., 2014). Eleven AOPs from AOP Wiki, pertaining to DART outcomes, were initially cited by Knapen et al. and since then others have been added for consideration (Knapen et al., 2015). However, many of these to a large extent still remain in development, limited by biological understanding and supporting evidence, and in some cases, a single AOP may not be sufficient to explain an AO and a network approach may be needed to explain or predict the final AO. A few examples are those of human hepatotoxicity, human neurotoxicity, swim bladder inflation in fishes, and male rat reproductive tract abnormalities, each of which are outlined by several distinct AOPs (Villeneuve et al., 2014; Spinu et al., 2019; Arnesdotter et al., 2021). Therefore, while the AOPs are very useful in development of NAMs, such an approach for even more complex outcomes like DART, especially in cases where the MoA of a chemical is unknown, is particularly challenging.

However, it is questionable whether prediction of specific adverse outcomes is necessary to enable decisions to be made on the safe use of chemicals. Do we need to be able to predict adverse DART outcomes (e.g., hypospadias, cleft palate, fused vertebrae), or is it more useful and relevant to know that under specified exposure conditions, an adverse DART outcome is not likely to happen? This change in mindset from a desire to *predict* adverse effects in high dose animal tests to an approach that seeks to *protect* humans from harm at relevant exposures provides the opportunity to allow context-dependent safety decisions to be made using the non-animal tools and approaches available today and to prevent unnecessary animal testing.

**FIGURE 1:**
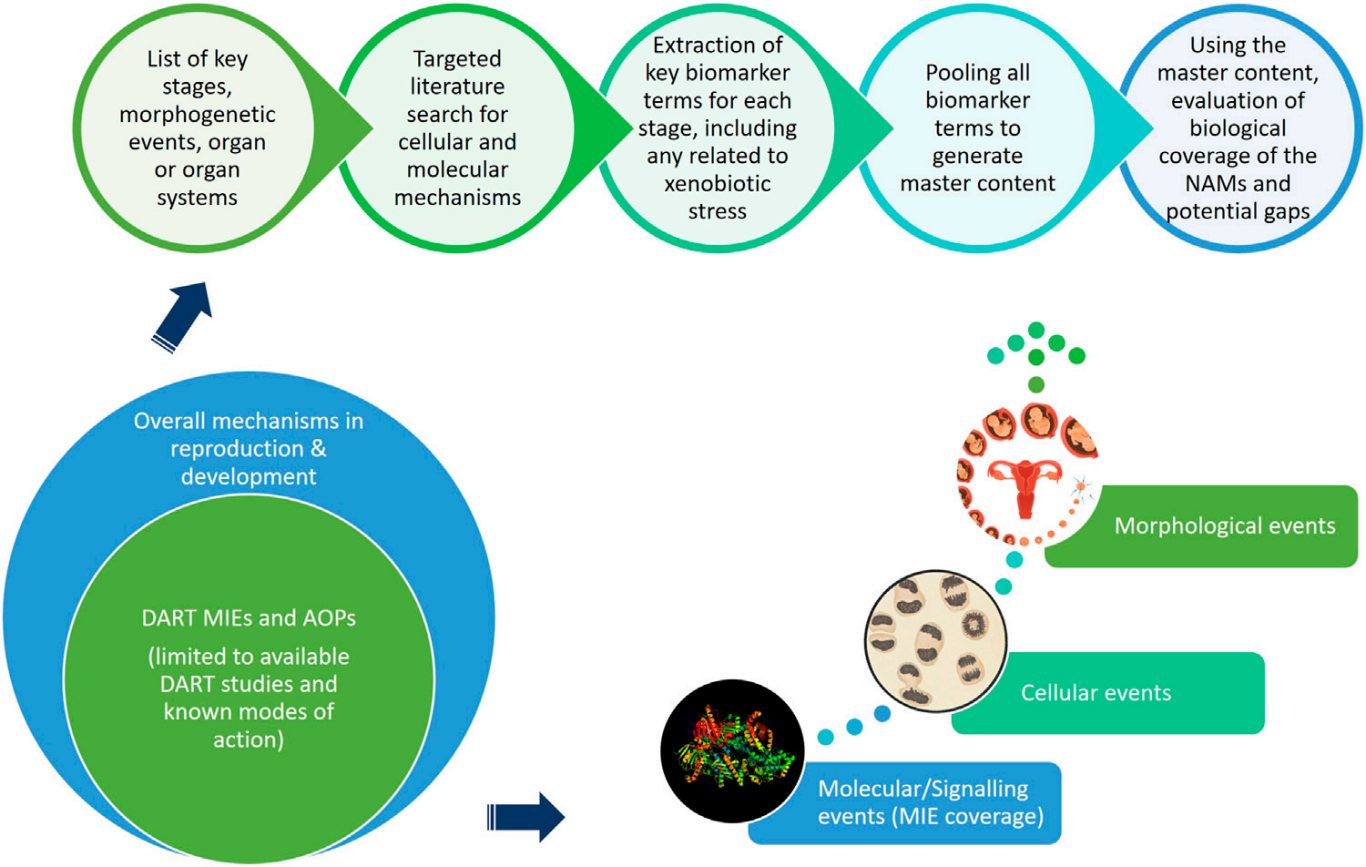
An approach using cellular and molecular information pertaining to reproductive and developmental biology to develop master list of significant biological markers.

**FIGURE 2:**
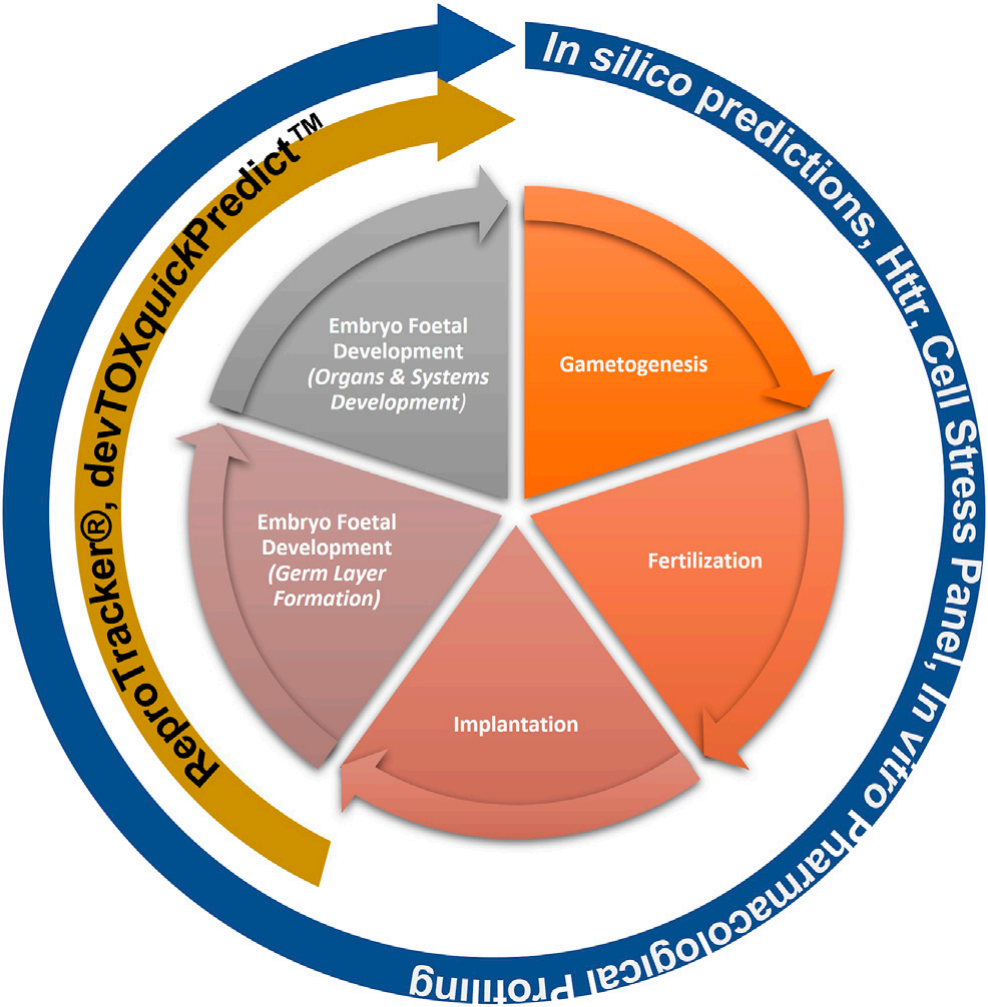
NAMs within the Developmental and Reproductive Safety Framework evaluated for being protective of DART effects spanning the key stages in reproduction and development.

This “protection, not prediction” philosophy set the context for the current work, which aimed to take advantage of the knowledge of mechanisms and signalling pathways reported to play a key role in human reproduction and embryo-foetal development, to evaluate how protective a DART NAM framework is for DART effects. As all morphological events or physiological processes are underpinned by cellular events and these are in turn orchestrated by molecular signalling events, gathering this cellular and molecular information pertaining to reproductive and developmental biology is a useful approach in developing a master list of biological markers of significance (Figure 1). A list of key stages and developmental landmarks, morphogenetic events, organ or organ systems was developed, followed by a systematic and targeted literature search conducted for cellular and molecular mechanisms underlying each of these stages. Relevant biological marker terms were then extracted from the abstracts of these publications, which were pooled to generate metadata of markers in human reproduction and development. Additionally, any markers related to general xenobiotic stress pathways and processes were also extracted into this metadata. This master content of biomarkers was used to evaluate the mechanistic coverage of the NAMs within our developmental and reproductive safety framework (Figure 2), assembled from the tools with the most biological coverage in the NGRA framework for systemic toxicity, outlined in Baltazar et al. (Baltazar et al., 2020), and newly available NAMs for detecting developmental toxicity. The overlay of the key set of markers upon the DART framework was also used to identify potential redundancies or gaps to be addressed. Finally, we exemplified our approach with a case study, where DART related NAM hazard data generated for caffeine is assessed against hypothetical exposure scenarios to explore how a framework could aid use of this information in decision making without generating any animal data.

## 2 Materials and Methods

### 2.1 Literature Search and Review

Guided by the overall knowledge of human reproductive biology and embryo-foetal development, the key stages and morphogenetic events, listed in Table 1, were considered for targeted literature search (Gilbert and Barresi 2016; Gabbe et al., 2017; Kliegman et al., 2020).

**TABLE 1:** Key stages, morphogenetics events and derivatives of human reproductive biology and embryo-foetal development.

Stage no	Key stages and morphogenetic events	Derivative organs and systems
1	Sex determination	
2	Gametogenesis	
3	Fertilization	
4	Zygote formation	
5	Implantation	
6	Blastulation	
7	Gastrulation	
8	Placenta formation	
9	Neurulation	
10	Ectoderm formation and its derivatives	a. Central nervous system
		b. Peripheral nervous system
		c. Autonomous nervous system
		d. Integumentary system
11	Mesoderm formation and its derivatives	a. Somitogenesis
		b. Hematopoiesis
		c. Heart and circulatory system
		d. Immune system
		e. Spleen
		f. Urinary system and urethra
		g. Reproductive system—testis
		h. Reproductive system—ovary
		i. Skeletal system
		j. Limbs
12	Endoderm formation and its derivatives	a. Digestive system
		b. Respiratory system
		c. Thymus
		d. Parathyroid
		e. Thyroid
13	Structures developing from mesenchyme or multiple germ layers	a. Adrenal glands
		b. Eyes
		c. Ears
		d. Face and neck
14	Intrauterine growth	

The EPA-developed Abstract Sifter literature review tool (https://github.com/USEPA/CompTox-Chemistry-Dashboard-Abstract-Sifter) was used to generate a set of relevant literature for each of the key stages listed above (Baker et al., 2017). In order to maximise the reports that either list or discuss biological markers, either related to signalling pathways or cellular processes, in the context of embryonic development, the query terms were standardised accordingly and aligned with the MeSH terms. Further, the focus of the literature search was on findings in human or mammalian systems and other vertebrate or invertebrate literature were excluded. Any reports related to infections were also excluded, due to limited value in understanding of developmental mechanisms. However, reports related to tumours or cancers were included as they can provide insights into normal developmental mechanisms indirectly. A standard query run was as follows:where x denotes specific terms related to each of the key stages and organs or systems from the master list. Additional inclusion terms related to specific physiology (e.g., nervous system or digestive system) or exclusion terms were used in a bespoke manner. Query terms used for each of the searches are provided in Supplementary File S1. To capture the use of different nomenclature describing the same term, index terms from the MeSH ontology were used, wherever possible, that included its synonyms, sub-tree terms and their synonyms. By using index terms, such as “fetal development” the search automatically expands to its synonyms (“fetal growth,” “fetal programming”), all sub-tree terms (“fetal movement,” “fetal organ maturity,” “fetal viability,” “fetal weight,” “gestational age”) and their synonyms.

“(x) AND (embryonic development OR fetal development) AND (cell physiology OR signalling OR pathway OR gene OR protein) AND (human OR mammalian) NOT (amphibians OR frog) NOT (fishes) NOT (invertebrates) NOT (birds OR aves OR avian OR chick) NOT (infections)”

Each set of the Abstract Sifter search results were saved using a filename indicating the respective stage or organ or system (Supplementary File S1). A quality check was performed manually to ensure the reports were relevant to the search criteria and minimise the number of unrelated results in cases when same terminology is used to describe different phenomena. This quality check review was conducted using the sifter terms functionality, where the key terms from the biology of interest were used (e.g., for central nervous system, the sifter terms included central nervous, spinal, neuron, neural, glial, astrocyte, etc.). Any report(s) that did not contain a single relevant key term but could not be excluded cleanly using specific exclusion terms were further highlighted for exclusion in the subsequent step of biomarker terms extraction.

### 2.2 Extraction of Key Biomarker Terms

Extraction of key biomarker terms was done using articles published in PubMed on or before 1 August 2021. For each stage/morphological event, related abstracts were first retrieved using relevant PubMed IDs (PMIDs) and then collated in a single text file. TERMite (https://www.scibite.com/platform/termite/), SciBite’s named entity recognition engine, was used to align unstructured text to extensively curated vocabularies, during a process of semantic enrichment. Semantic enrichment was limited to three vocabularies, namely, GENEBOOST, miRNA and DrBP (DART-related Biological Processes).

GENEBOOST is SciBite’s vocabulary based on the Hugo Gene Nomenclature Committee standard list of genes that covers all human protein coding genes (Tweedie et al., 2021; DOI:10.1093/nar/gkaa980).

miRNA is SciBite’s algorithmic module that detects miRNA terms present in the text.

DrBP is a bespoke vocabulary that was generated for the purpose of this work. The vocabulary includes relevant cellular and molecular mechanisms that underly each of the stages pertaining to reproductive and developmental biology. Specifically, the vocabulary was formed by merging terms relevant to signalling pathways and cellular processes contained in the publicly available Gene Ontology (GO) and National Cancer Institute Thesaurus (NCIT) (Ashburner et al., 2000) (https://ncit.nci.nih.gov/ncitbrowser/). Namely, it contains: 1) Cell Stress Process class from NCIT (id: C21065) that includes cellular or subcellular processes involved in disturbance or restoration of a homeostatic condition; 2) Epigenetic Process class from NCIT (id: C21051) that includes terms related to changes in the regulation of the expression of gene activity without alteration of genetic structure; 3) Mitochondrial Damage class from NCIT (id: C45524) that includes any process that leads to dysfunction of mitochondria, whether by oxidative damage, mutation of mitochondrial DNA or other by means; 4) Cellular Process class from GO (id: 0009987) that includes any process that is carried out at the cellular level, but not necessarily restricted to a single cell; 5) Signalling class from GO (id: 0023052) that includes processes in which information is transmitted within a biological system.

For the terms extracted using GENEBOOST and DrBP alone, a threshold of ≥10 and ≥5 hit counts, respectively, was decided to maximise the relevance of the extracted key biomarker terms. For the terms extracted using the miRNA module, this threshold was set to at least two appearances of a relevant miRNA term in all abstracts associated with the specific key stage/morphological event from Table 1.

## 3 Biological Coverage of NAMs

### 3.1 Baseline Gene Expression of MCF7, HepG2 and HepaRG Cells in High Throughput Transcriptomics (HTTr)

The 3 cell lines that are currently core to our systemic toolbox are MCF-7, HepG2 and HepaRG cells. To enable a comparison between the key biomakers identified above using GENEBOOST and the coverage provided by these cell lines, baseline gene expression for each cell line was obtained by analysing gene counts for 24 h with 0.5% DMSO as a vehicle control. The data analysis pipeline followed Reynolds et al. (Reynolds et al., 2020). According to Reynolds et al., probes with a mean or median count less than five across all non-control treatments were discarded. Applying this procedure, we have created three separate gene lists that establish a baseline expression for each of the cell line used in the experiments (Supplementary File S2).

### 3.2 Baseline Gene Expression of iPSCs

The Stemina DevToxQuickPredict and Toxys ReproTracker assays use human induced pluripotent stem cells (iPSCs). An example of baseline gene expression of iPSCs in undifferentiated state was obtained by analysing previously deposited gene expression of eight human iPSCs from StemCellDB (Mallon et al., 2013). The dataset represents quantile normalized log (base = 2) gene expression data from Agilent one-color gene expression microarrays. According to Mallon et al., a gene was considered expressed in this data set if it was detected in at least one of the eight iPSCs using a noise cut-off of 7.5. Applying this procedure, we have created a gene list which defines pluripotency and establishes a baseline for the undifferentiated pluripotent state (Mallon et al., 2013) (Supplementary File S3), which was then utilized to compare with the key biomarkers extracted with GENEBOOST.

### 3.3 *In Vitro* Pharmacological Profiling (IPP)

The *In vitro* Pharmacological Profiling (IPP) panel contains 72 binding, enzymatic and coactivator recruitment assays associated with 60 targets with known safety liabilities. 44 of the targets have been previously associated with *in vivo* adverse drug reactions (Bowes et al., 2012). An additional 16 targets implicated in developmental toxicity were added to the panel based on available literature (National Research Council et al., 2000; Wu et al., 2013). They include six nuclear hormone receptors (Estrogen Receptor Alpha, Estrogen Receptor Beta, Thyroid Hormone Receptor Alpha, Thyroid Hormone Receptor Beta, Progesterone Receptor and Mineralocorticoid Receptor) and 10 basic helix-loop-helix transcription factors (Retinoic acid receptor alpha, Retinoic acid receptor gamma, Retinoic acid receptor RXR-alpha, Retinoic acid receptor RXR-beta, Peroxisome proliferator-activated receptor alpha, Peroxisome proliferator-activated receptor delta, Peroxisome proliferator-activated receptor gamma, Pregnane X receptor, Constitutive androstane receptor, Vitamin D Receptor). The full list of the IPP targets (Supplementary File S4) was compared with the list of biomarkers identified using DrBP.

### 3.4 Cell Stress Panel (CSP)

Cell stress panel comprises biomarkers that cover eight key stress pathways (oxidative stress, inflammation, ER stress, metal stress, DNA damage, heat shock response, hypoxia and translocation of Aryl Hydrocarbon Receptor), mitochondrial toxicity and general cell health (Simmons et al., 2009). For more information on the specific list of assays included in the panel refer to (Hatherell et al., 2020). The CSP assays were compared with the list of biomarkers identified using DrBP.

### 3.5 devTOXquickPredict^™^

devTOX*quick*Predict^™^ is a targeted exposure-based biomarker assay on human embryonic stem cells (hESCs) or induced pluripotent cells (iPSCs), where in response to a nine-point dose response testing, specific metabolites, along with a cytotoxicity endpoint, are monitored. Specifically, the ratio of ornithine to cystine is measured as they were identified as indicators of developmental toxicity (Palmer et al., 2013; Palmer et al., 2017). This output was used to assess the coverage of certain specific cellular processes identified by DrBP.

### 3.6 ReproTracker^®^

ReproTracker^®^ assesses any perturbation, following chemical exposure, to the process of differentiation of stems cells to specific germ layers and the derivatives therein, by monitoring specific biomarkers (Racz et al., 2018) (Jamalpoor et al., submitted 2021). Human induced pluripotent stems cells (hiPSCs), differentiating into endoderm, mesoderm or ectoderm cell types and further to derivative hepatocytes, cardiomyocytes and neural rosettes, respectively, were treated with six different doses of the test chemical. Any change in levels of AFP, MYH6 and Pax6 expression as well as other morphological features were monitored, as markers for the differentiation of liver, heart and neural cells, respectively, and thereby potential teratogenic effects were determined. These read-outs were compared with the key biomarkers extracted by DrBP, specifically those related to differentiation processes.

### 3.7 *In Silico* Predictions

Based on accurate representations of chemical structure, *in silico* predictions can be used to inform on the potential biological activity profile of the molecule, give indication of any possible selective or non-selective activity and provide a broad toxicity screen across many apical endpoints based on the existing toxicity data. These predictions were used as weight of evidence for biological coverage. For more information on the list of *in silico* tools used as a part of the DART Toolbox and their associated endpoint refer to Supplementary Table S3.

## 4 Development of a NGRA DART Framework and Application to the Risk Assessment of Caffeine in Consumer Products

**FIGURE 3:**
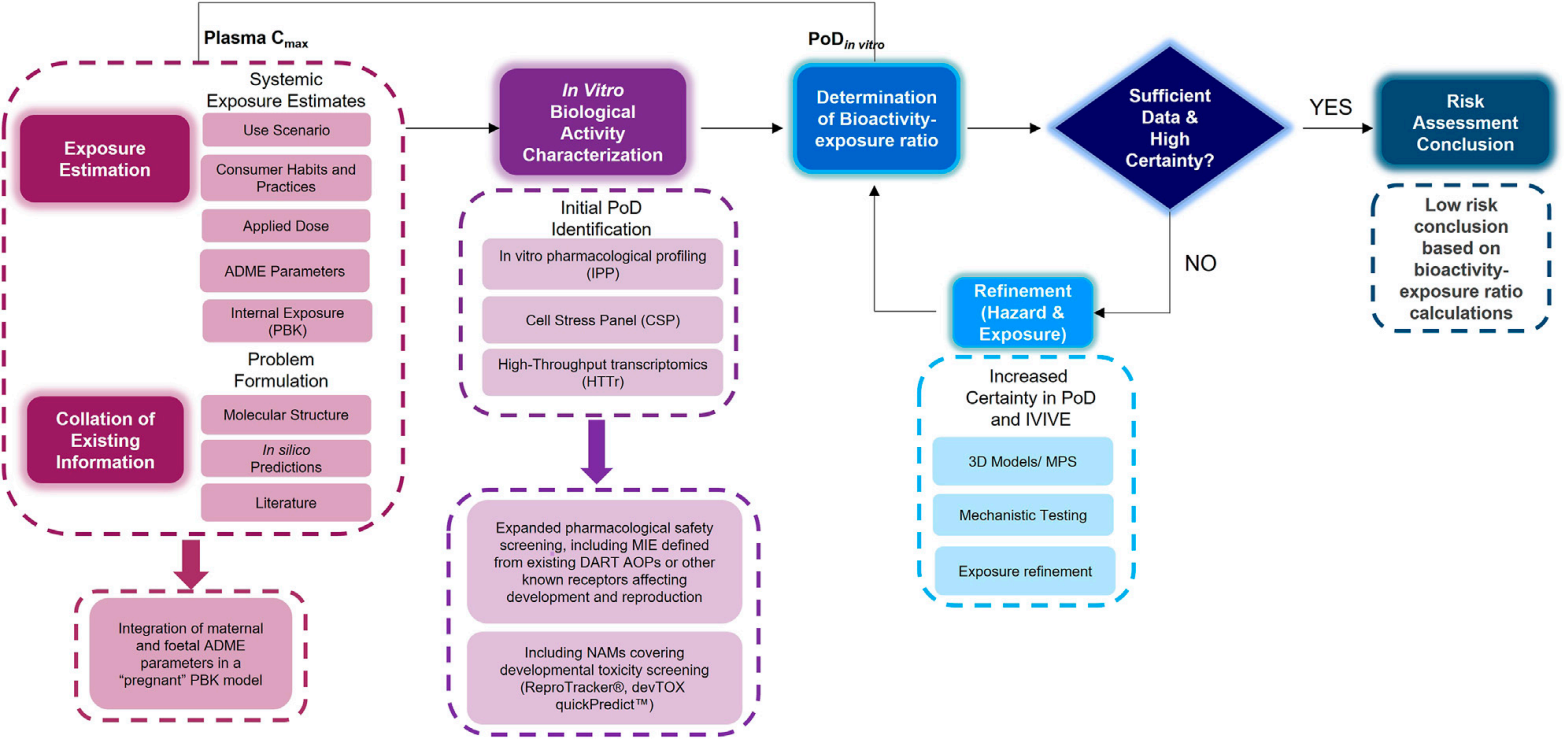
An NGRA framework outlining the consideration of any existing information with exposure estimation including maternal and foetal ADME parameters with *in vitro* biological activity characterisation including additional NAMs relevant for DART endpoints to determine the bioactivity exposure ratio and further refinements to arrive at a risk assessment conclusion.

An NGRA workflow, illustrating how to integrate NAMs for the systemic safety assessment of coumarin has been previously published (Baltazar et al., 2020). Despite the workflow being tailored to the coumarin case study, its key building blocks are applicable to any NGRA. We hypothesize that the NAMs described in Figure 2 could form a core toolbox within an NGRA framework which is protective of DART effects (Figure 3). If there is sufficient confidence that this toolbox provides coverage for the processes that are critical for normal reproduction and development, the bioactivity-exposure ratio (BER) (ratio between PoD and exposure) can be calculated and, together with all the other available evidence a decision can be made to whether there is sufficient information and high certainty to reach a risk assessment conclusion. If the outcome is uncertain, an iterative process can begin by designing new experiments addressing specific gaps (Figure 3).

Caffeine was selected as a case study chemical to exemplify the use of the DART framework, based on the availability of human exposure data from cosmetics and dietary sources and known mode of action. High consumption of caffeine (>200 mg/day) during pregnancy has been associated with increased risk of adverse birth weight-related outcomes (i.e., foetal growth retardation, small for gestational age), and therefore the European Food Safety Authority (EFSA) has advised a limit of 200 mg/day for pregnant women instead of the 400 mg/day for the general adult population (EFSA, 2015). The objective of this case study was to investigate how protective our NGRA DART framework is for chemicals suspected of causing reproductive and developmental adverse health effects in humans. To exemplify how this can be achieved, two distinct exposure scenarios were selected, a dermal exposure to 0.1% caffeine in a hypothetical body lotion and an oral consumption of 200 mg/day of caffeine at different gestational stages of pregnancy (6, 20 and 30 weeks). After determining exposure, *in silico* tools (see *In Silico* Predictions) were run followed by the generation of the *in vitro* biological activity data, including the IPP, CSP, HTTr, ReproTracker^®^ and devTOXquickPredict™. PoDs derived from the CSP, HTTr and IPP were compared to exposure estimates (maternal and foetal plasma C_max_) to calculate a bioactivity-exposure ratio (BER).

### 4.1 Exposure Estimation: PBPK Modelling

#### 4.1.1 Workflow

A PBK model was built to make predictions on both maternal and foetal concentration (i.e., plasma C_max_) of caffeine under two exposure scenarios, i.e., oral administration of 200 mg caffeine and topical application of a hypothetical body lotion containing 0.1% caffeine, so that the C_max_ concentrations could be compared against *in vitro* PoD values to derive the BER. Although foetal concentration is a more relevant dose metric for a substance suspected of being a direct developmental toxicant, both maternal and foetal concentrations were predicted throughout different trimesters in pregnancy, and C_max_ from all the predicted maternal and foetal concentrations were used in the BER calculation to be conservative in decision making.

**FIGURE 4:**
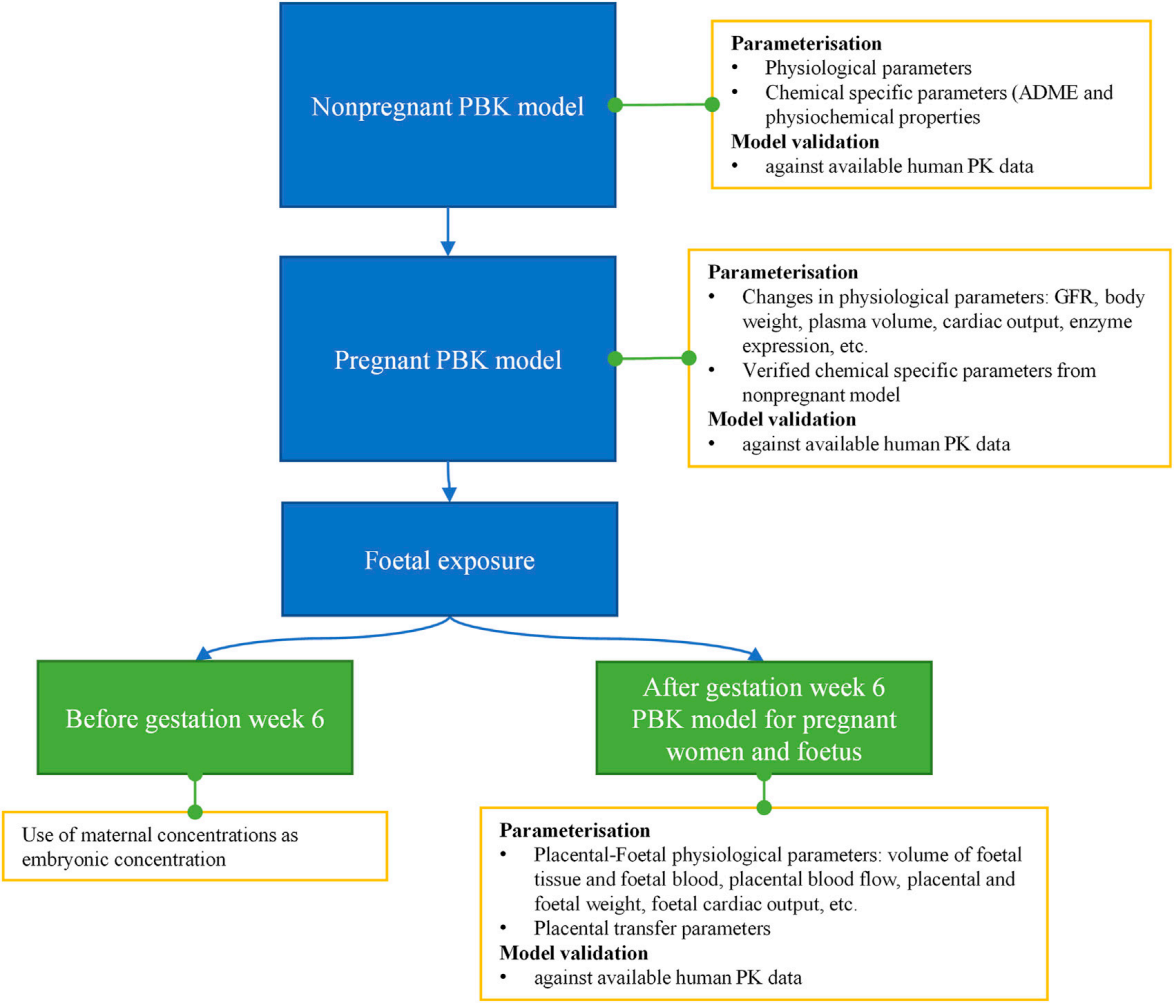
Schematic diagram of the workflow of pregnant PBK model development for predicting foetal exposure.

The workflow we used to develop the PBK model is shown in Figure 4. Firstly, a non-pregnant PBK model was built and verified against available observed human clinical data from both oral and dermal exposures routes. In case the predicted PK profile and parameters deviated from the observed data, the model was refined by parameter optimization through fitting against the non-pregnant clinical data. Secondly, the pregnancy PBK model was developed with the verified chemical-specific parameters and pregnancy-related physiological changes over time, and then further validated against available human PK data in pregnancy. Thirdly, the verified pregnancy PBK model was extended for embryonic/foetal concentration prediction at different gestational ages. In the foetal period of development, foetal circulation is established during the early stages (week 5–6 of gestation), allowing the growing fetus to receive the required oxygen and nutrients as well as dispose of waste products. This type of circulation involves the umbilical cord and placental blood vessels which carry foetal blood between the foetus and the placenta (Wang and Zhao, 2010). Therefore, in the pregnancy PBK model, week 6 of gestational age (GA) was chosen to separate two stages. For GA before week 6, only the uterus tissue is added to the model; for GA above week 6, in addition to the uterus tissue, placenta tissues, amniotic fluid, foetal tissue and foetal blood circulation are also included in the model. Lastly, the extended pregnancy PBK model was used to make predictions on plasma concentrations in both mother and embryo/foetus at different GAs under the two exposure scenarios of interest.

#### 4.1.2 Software

PBK models for caffeine were built using the software GastroPlus 9.8 (Simulation Plus, Lancaster, CA, United States). Population-dependent physiological parameters for non-pregnant and pregnant PBK models were obtained using the Population Estimates for Age-Related (PEAR) Physiology module in GastroPlus. Pregnancy incurred changes in the maternal body: weight gain, changes in enzyme expression levels, enlargement of certain tissues such as uterus, placenta, brain and kidney, increased amount of plasma volume, GI changes (increased stomach transit time), etc., as wells as the volume of foetal tissue and foetal blood with GA have all been incorporated in the built-in pregnancy model. When maternal age, GA and foetal gender are specified, the default physiology will be generated by built-in equations. Alternatively, the default physiologies for both maternal and foetal subjects, such as body weight and height, cardiac output, weight gain and individual tissue weights and perfusions could be manually modified.

#### 4.1.3 Development and Validation of the Non-Pregnant and Pregnant PBK Model

The physiochemical and ADME parameters used to build the PBK models were derived from published *in silico*, *in vitro*, and human PK data, and are presented in Supplementary Table S1. Tissue-to-plasma partitioning coefficients were calculated in Gastro-Plus using the Lukacova (Rodgers–Single) method, assuming chemical distribution into the tissues being perfusion-limited. The liver and kidney were considered to be the only organs to eliminate caffeine. Caffeine is mainly metabolized by CYP1A2 (Grzegorzewski et al., 2021). The liver metabolism was described in the model by using *in vitro* Km and V_max_ values for CYP1A2 reported in the literature, and that the V_max_ was then optimized by GastroPlus to better fit caffeine plasma profiles and predict clearance in non-pregnant clinical data of intravenous infusion (Blanchard and Sawers 1983; Cheng et al., 1990; Otberg et al., 2008). The model was then validated by comparing the simulated PK data with the observed clinical studies from both oral and dermal applications (Blanchard and Sawers 1983; Cheng et al., 1990; Otberg et al., 2008). After the non-pregnant PBK model was developed and verified, the pregnancy PBK model was built with the same organs mentioned in non-pregnant subjects, the verified chemical-specific parameters and pregnancy incurred physiological changes (the physiological changes have been incorporated in GastroPlus for the pregnancy population). It is worth noting that the decrease in CYP1A2 activity for each pregnancy trimester was captured in the model too. Then this pregnancy PBK model was validated by comparing the simulated PK data with the available observed clinical study (Brazier et al., 1983).

#### 4.1.4 Extending the Pregnant PBK Model for Embryonic/Foetal Exposure Prediction

The transfer of substances between the maternal to foetal blood-stream comprises of materno and foetosyncytiotrophoblast exchanges at the apical and basal face of the syncytiotrophoblast, respectively (Codaccioni et al., 2019). Compounds transfer across the placental *via* passive diffusion, active transport, facilitated diffusion, pinocytosis and phagocytosis. Passive diffusion and active transport, to a lesser extent, are the predominant mechanisms of placental transfer for small molecules, like caffeine (Griffiths and Campbell 2014). In this study, passive diffusion was considered to be the main mechanism for placental transfer of caffeine, as no evidence has been shown that it undergoes active uptake/efflux in placenta. Unlike other tissues, placenta was set to be permeability limited tissue in the pregnancy model. The placental transfer input, i.e., apparent permeability coefficient (Pe) was obtained from *in vitro* placental transfer assays using BeWo b30 cells as reported in literature (Supplementary Table S1). This parameter was needed to calculate the PStc (permeability cellular surface area product) in the placental compartment. The cellular surface areas of placenta at different GA were obtained as reported in literature (Griffiths and Campbell, 2014; Dhyani, 2021).

#### 4.1.5 PBK Predictions on Maternal and Foetal Concentrations of Caffeine Under Two Exposure Scenarios

The extended pregnancy PBK model was used to make predictions on plasma concentrations in both mother and embryo/foetus at different GAs under the two exposure scenarios of interest. The simulation of each scenario was run to predict steady state concentration profiles on 30 years old pregnant women. For the topical application, consumer exposure to body lotion was characterised based on SCCS guidance as presented in Supplementary Table S2 and used in the dermal module in GastroPlus as product use input.

### 4.2 Collation of Existing Information

Caffeine (CAS: 58-08-2) is a member of the methylxanthine group, commonly found in foods and drink. The suite of *in silico* tools described above was used to provide predictions of toxicity based on caffeine’s structure (Supplementary Table S3). For completeness, tools were run for non-DART toxicological endpoints in addition to the DART relevant endpoints.

### 4.3 *In Vitro* Biological Activity Characterisation

*In vitro* biological activity characterisation was performed by generating data for caffeine in the following tools: CSP, HTTr, IPP, ReproTracker^®^ and devTOXquickPredict™ and methods are summarised below.

Caffeine was tested in cell stress panel according to Hatherell et al. (Hatherell et al., 2020). Concentration-response datasets were analysed with a Bayesian statistical model which infers a distribution for the PoD. The cumulative density function of the distribution, evaluated at a chosen concentration, is regarded as a measure of confidence in perturbation of the measured biomarker at the chosen concentration. Confidence at the maximum tested concentration was reported as the *concentration-dependency score (CDS).*

Caffeine was tested in multiple cell models in two different experiments using high-throughput transcriptomics TempO-Seq technology (Yeakley et al., 2017; Baltazar et al., 2020). Experiment 1 included MCF7, HepG2 and HepaRG 2D cells and in experiment 2 HepaRG 3D model was included as well as a repeat in HepaRG 2D cells. Cells were cultured following procedure from Baltazar et al. Concentrations were chosen after an initial cytotoxicity study to exclude cytotoxic doses (if present) and the dose range chosen to encompass the two exposure scenarios (0.01, 0.1, 1, 10, 100, and 1,000 µM). Cells were treated for 24 h using 0.5% DMSO as a vehicle control. Three biological replicas were generated for the experiment 1, whereas five biological replicas were generated for the experiment 2. Gene sequencing was performed on cell lysates using the Biospyder Human Whole Transcriptome Assay panel version 1 as previously described by (Yeakley et al., 2017). The data processing pipeline and concentration–response analysis followed a modification of the method developed by Reynolds at al (Reynolds et al., 2020). Briefly, a Bayesian statistical model similar to that developed in (Baltazar et al., 2020) was constructed to derive PoDs for individual probes. Modifications of the analysis method include the use of a log Student’s t-Poisson sampling distribution rather than a negative binomial to allow for increased kurtosis in the distribution of counts, thereby reducing sensitivity to outliers. In addition, variance across biological replicates was no longer used to determine the PoD to increase sensitivity. Lastly, the global PoD which is an estimate of a minimum effect concentration across all probes [see (Reynolds et al., 2020) for further details], is calculated from quantiles in the range 25–75 rather than 1–99 to increase stability of the estimate.

IPP experiments were carried out at Eurofins Cerep SA. At first, the screening was done using a fixed concentration of caffeine at 10 uM in two replicates. Compound binding in binding assays was calculated as a percentage inhibition of the binding of a radioactively labelled ligand specific for each target. Compound enzyme inhibition effect was calculated as a percentage inhibition of control enzyme activity. In order to determine IC50 (concentration causing a half-maximal inhibition of the control response) as a measure of potency for the Adenosine 2A receptor inhibition, the assay was performed in a dose response manner at eight concentrations in two replicas. The IC50 value was determined by the Bayesian probabilistic model of the concentration-response curve (Johnstone et al., 2017). The priors for IC50 were set to the median experimental dose, the slope was set to 1.0 and low and high dose responses were set to 0% and 100%, respectively.

Results for caffeine exposure in the devTOXquickPredict™ assays were extracted from Zurlinden et al. In short, H9 cells were treated with up to 500 μM caffeine for 72 h, with media replacement every 24 h. The cell-conditioned media from the final 24 h treatment period was collected for analysis of the targeted biomarker, ornithine and cysteine ratio, and cell viability (Zurlinden et al., 2020). For the ReproTracker^®^, hiPSCs were differentiated into hepatocytes, cardiomyocytes and neural rosettes, respectively, in the presence of six doses of caffeine (up to 100 μM), for 21, 14 and 13 days, respectively, and any change in levels of AFP, MYH6 and Pax6 expression as well as other morphological features were monitored.

### 4.4 Determination of Bioactivity Exposure Ratio (BER)

For a given PoD, the BER was defined as the ratio between the nominal concentration at which the PoD is defined, and the relevant plasma Cmax estimate from the PBPK modelling for both exposure scenarios. PoDs were derived from the HTTr, Cell Stress and iPP panel.

## 5 Results

### 5.1 Literature Search and DARS Markers Extraction

**FIGURE 5:**
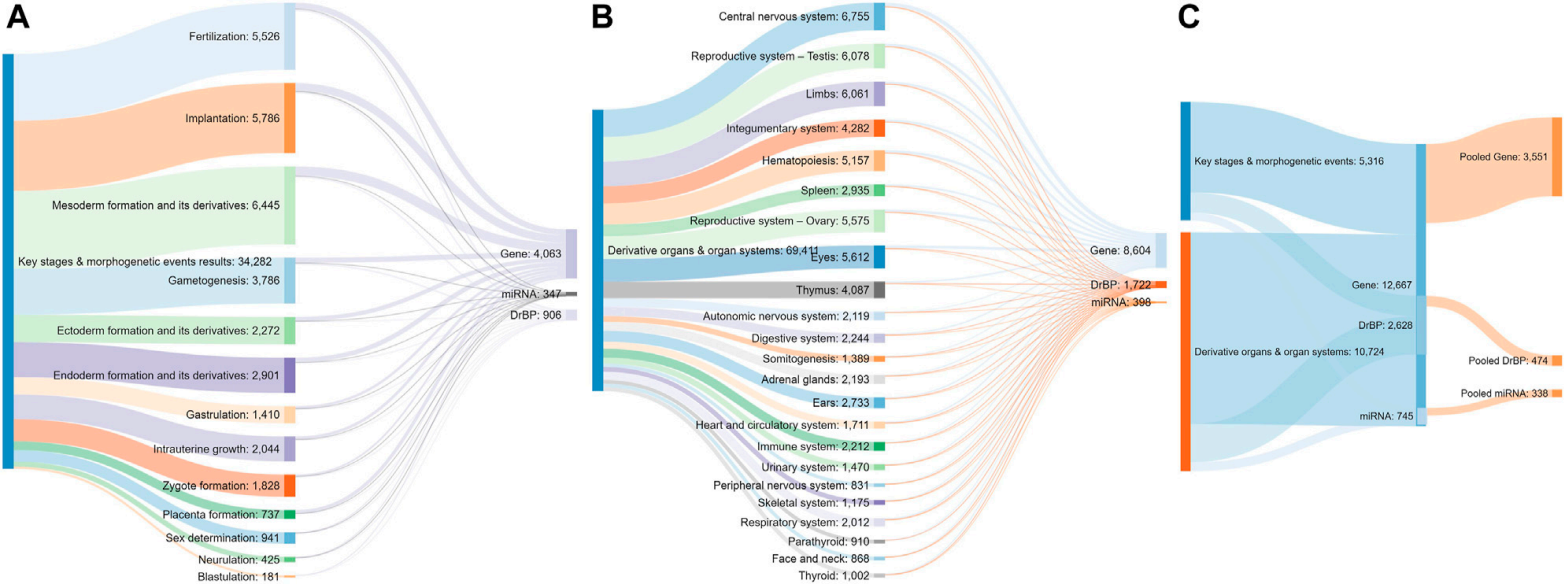
Sankey diagram indicating the number of articles screened for each stage **(A)** and organ type **(B)**, the number of stage **(A)** or organ-specific **(B)** sets of DARS markers extracted and pooled sets of DARS markers **(C)**.

A total of 34,308 articles from the literature search on key stages in reproductive biology and embryo-foetal development as well as morphogenetic events (Table 1) were found to be relevant, after validating the results through sifter criteria. Similarly, a total of 69,299 relevant articles were short listed from the searches related to organs and organ systems development (Table 1). This complete list of articles is provided in Supplementary File S1. A certain degree of overlap in articles related to each of the germ layers and their derivatives was seen. However, all the relevant articles were considered for the next steps as an over representation of information was preferred rather than an under representation. The pooling of extracted markers in the subsequent steps addressed any duplications and redundant information. These 103,607 articles served as the comprehensive pool from which biological marker terms relevant to signalling during reproduction and development, referred to as Developmental and Reproductive Signalling (DARS) markers, were extracted (Figure 5). The overall process is depicted in Figure 1.

A named entity recognition engine Termite (SciBite) was used to align the titles and abstracts of the pooled articles to extensively curated vocabularies that represent human genes, cellular and signalling processes, miRNAs and their synonyms (see *Methods* for more information). This enabled recognition and annotation of multiple terms that are used to describe the same concept (e.g., gene aliases, alternative pathway names, etc). A quality check was performed to ensure the extracted terms are indeed relevant and do not refer to any other meaning. The final list of DARS markers that refer to genes, cellular/signalling processes and miRNAs extracted for each key stage is given in Supplementary Files S5–S7, respectively.

**FIGURE 6:**
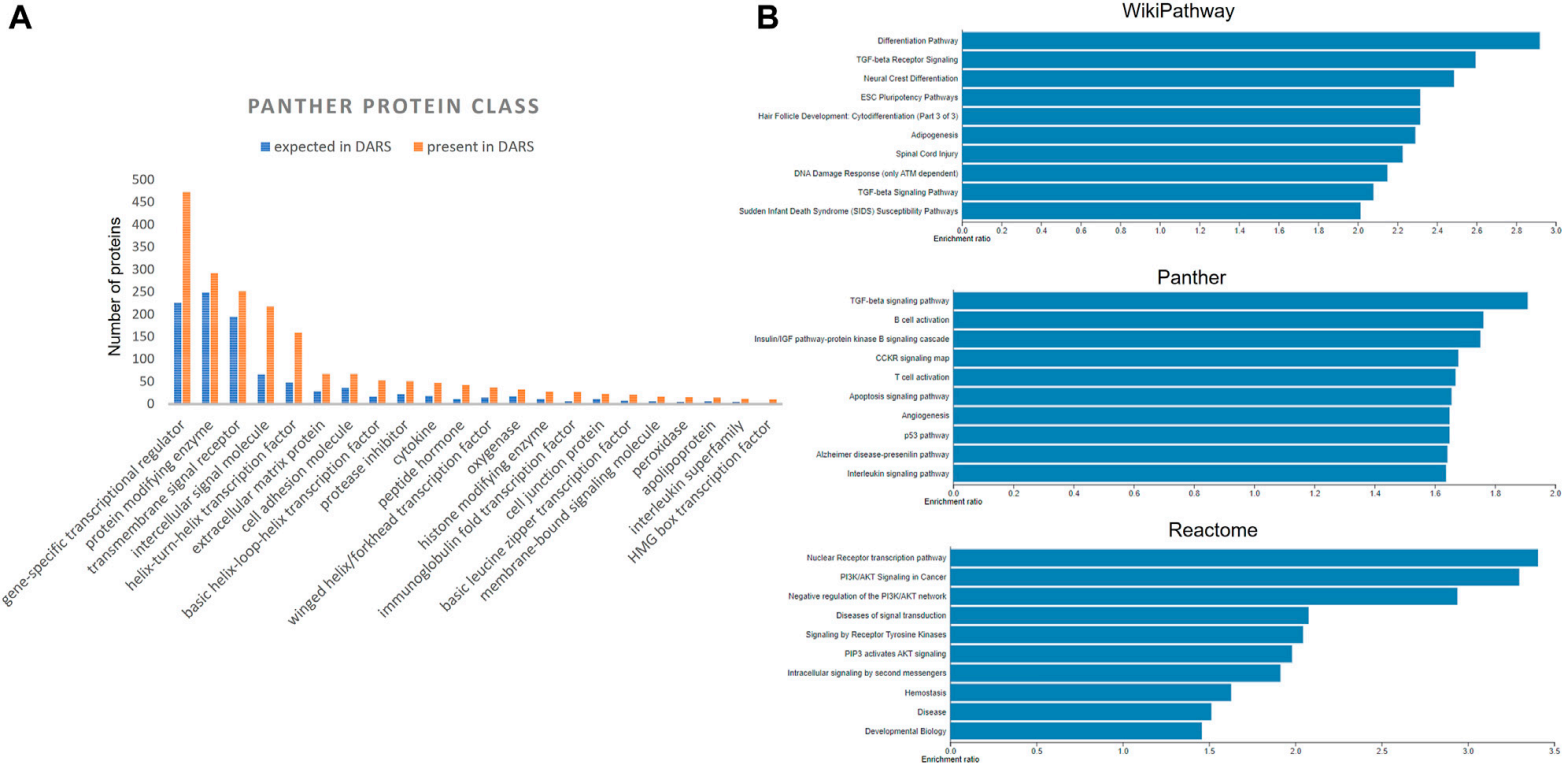
**(A)** Distribution of DARS genes across the Panther protein classes **(B)** Over-represented pathways involving DARS genes from WikiPathways, Panther and Reactome, analysed using WebGestalt.

Extracted DARS genes are presented separately for each stage in the development (Supplementary File S5). For instance, they included 902 DARS genes associated with the development of the central nervous system or 785 DARS genes associated with the mesoderm formation. Furthermore, additional validation was carried out by checking if DARS gene sets extracted for each stage were indeed related to the stage in question. This was done by annotating extracted DARS gene sets using the GO biological processes and Human Phenotype Ontology. Then, for each key stage top 10 statistically overrepresented GO biological processes and phenotypic abnormalities were reviewed and their relevance to the key stage was confirmed (Supplementary File S5). For example, in the gene set associated with the development of the central nervous system, processes and abnormalities such as neuron migration, regulation of neurotransmitter levels, *in utero* embryonic development, neoplasm of central nervous system, neoplasm of the nervous system and morphological abnormalities of the central nervous system were among the top overrepresented, which supports the conclusion that the associated DARS gene set is indeed very relevant. Lastly, gene sets for each stage in the development were pooled together in a final list of 3,551 DARS genes (Figure 5, Supplementary File S5). Three genes with the highest number of occurrences in the literature were found to be glycoprotein hormones alpha chain (11,924 hits), sonic hedgehog protein (6,622 hits), and proto-oncogene Wnt-1 (6,428 hits). Glycoprotein hormones alpha chain encodes for the alpha chain of the active heterodimeric glycoprotein hormones, such as thyroid stimulating hormone, luteinizing hormone, follicle stimulating hormone and choriogonadotropin. Sonic hedgehog and Wnt-1 are well-known signalling proteins that play essential role in developmental processes. When the 3,551 DARS genes were classified using protein classes from Panther (Mi et al., 2019), gene-specific transcription regulators, protein modifying enzymes, transmembrane and intercellular signal molecules came up as top four overrepresented classes (Figure 6A). In addition, the top overrepresented pathways, analysed using WebGestalt server (Zhang et al., 2005), from WikiPathways (Martens et al., 2020), Panther (Mi et al., 2019) and Reactome (Jassal et al., 2020) were found to be the differentiation pathway, TGF-beta signalling, and nuclear receptor transcription pathway, respectively. For the list of top 10 overrepresented pathways from the three databases refer to Figure 6B.

DARS cellular and signalling processes mined in the literature are presented separately for each stage in the development (Supplementary File S6). For instance, they included 145 DARS cellular and signalling processes associated with the development of central nervous system or 114 processes associated with the mesoderm formation. When individual processes for each stage in the development were pooled together, they gave a final list of 474 DARS processes (Figure 5, Supplementary File S6). Among them, signalling was by far the most mentioned term in the literature with 21,733 occurrences. This was followed by cell cycle and cell death with 3,228 and 2,514 hits, respectively. Other top 10 processes included DNA methylation (2,440 hits), epithelial to mesenchymal transition (2,422 hits), phosphorylation (2,372 hits), cell differentiation (2,262 hits), cell development (2,248 hits) and oocyte maturation (1,973 hits); all of them known to be cardinal in reproductive and developmental biology.

Lastly, DARS miRNAs mined in the literature are presented separately for each stage in the development (Supplementary File S7). The number of miRNAs associated with different stages showed high variability from 90 miRNAs associated with the implantation process to no miRNAs detected for the blastulation and development of parathyroid and urethra (see Supplementary File S7 for all key stages). Furthermore, additional validation was attempted by checking if targets associated with the miRNAs sets extracted for each stage were indeed related to the stage in question by using miRNA-target annotation available in the IPA software (Kramer et al., 2014). However, we were not able to confirm any overrepresented GO biological processes or phenotypic abnormalities that would confirm their relation to different stages in the development. MiRNAs have previously been shown to have diverse roles in fundamental biological processes such as cell proliferation, differentiation, apoptosis and stress response and are important regulators in development [reviewed in (Shenoy and Blelloch, 2014; Ivey and Srivastava, 2015)]. While the research on miRNA is steadily increasing, the annotation of miRNAs itself as well as to its biological function is only emerging (Huntley et al., 2018; Kozomara et al., 2019; Alles et al., 2019) which might explain the unsuccessful search in IPA. However, we still believe it is useful to report here the list of DARS miRNAs found to be associated with the development from the literature. When individual miRNA sets for each stage in the development were pooled together, they gave a final list of 338 DARS miRNAs (Figure 4, Supplementary File S7). Top three miRNAs were the let-7 family, miR-21 and miR-145 with 155, 127, and 85 occurrences in the literature, respectively. They have all been previously reported to play an important role in reproductive and developmental biology; the let-7 family is well-known regulator of iPSC reprogramming that promotes differentiation, miR-21 plays a role in cell proliferation, migration, invasion and apoptosis, whereas miR-145 was found to regulate differentiation of multipotent neural crest cells (Ji et al., 2007; Worringer et al., 2014; Sekar et al., 2019). As miRNAs regulate mRNA levels and they form a set of complementary biomarker data to that of gene expression, detailed correlation between the two in a spatial and temporal context would be required to outline their coverage and was decided to be out of scope for this work. For this piece of work, we have focussed on the coverage of transcriptomic markers in greater detail.

### 5.2 Biological Coverage of DARS Markers

Building on the previously published framework for evaluating systemic toxicity in a safety assessment, which included read-outs from *in silico* predictions, HTTr, cell stress panel and IPP (Baltazar et al., 2020), we developed an NGRA DART framework which includes two additional NAMs relevant for DART endpoints, namely, ReproTracker^®^ (Racz et al., 2018) (Jamalpoor et al., submitted 2021) and devTOXquickPredict™ (Palmer et al., 2013) (Figure 2). In order to assess the coverage of human biology and mechanistic relevance of our NGRA DART framework, we set out to evaluate the coverage of individual NAMs integrated in this framework against the identified DARS markers.

#### 5.2.1 Genes

**FIGURE 7:**
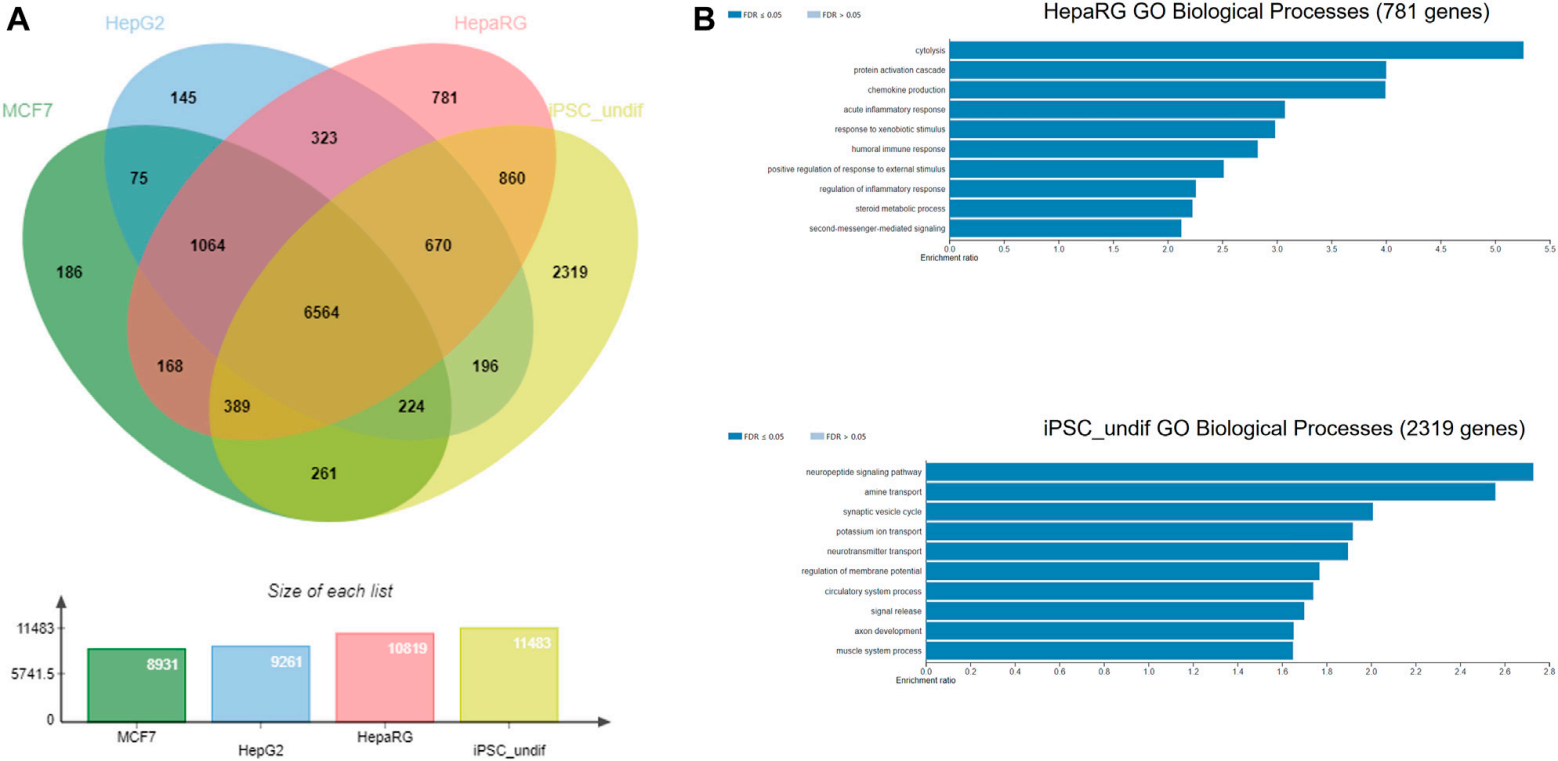
**(A)** Gene coverage across MCF-7, HepG2, HepaRG and undifferentiated iPSC cell lines **(B)** Over-represented processes exclusive for HepaRG and iPSC cells, analysed using WebGestalt.

The NGRA framework routinely employs MCF7, HepG2 and HepaRG cell lines for HTTr testing and caffeine was also tested in these 3 cell lines. Hence, as an example of biological coverage of the HTTr assay we identified separate gene lists that established a baseline expression for each of the cell line used in the experiments (see Methods and Supplementary File S2). They include 8,931 genes expressed in MCF7, 9,261 genes expressed in HepG2s and 10,819 genes expressed in HepaRG cells. In addition, an example of baseline gene expression of human iPSCs in undifferentiated state was obtained by analysing previously deposited gene expression in eight human iPSCs from StemCellDB (Mallon et al., 2013). Specifically, they include 11,483 genes with details presented in the Supplementary File S2. This analysis is used to approximate and inform on the coverage of biology of the devTOXquickPredict™ assay, which also uses iPSCs in the undifferentiated state. Genes detected in MCF7, HepG2, HepaRG, iPSC cells were pooled together, and their overlap is depicted in Figure 7. They include 14,225 genes in total. Almost half of those genes (6,564 genes) were found to be expressed in all four cell lines. Among the cell lines, iPSCs express 2,319 genes that are not present in any of the remaining cell lines referred to as unique genes here; HepaRGs, MCF7s and HepG2s express 781, 186 and 145 unique genes, respectively (Figure 7A). Some of the GO Biological Processes overrepresented in the genes unique to iPSC include neuropeptide signalling pathway, regulation of membrane potential, axon development and muscle system process (Figure 7B). Cytolysis, acute inflammatory response, and response to xenobiotic stimulus are some of the processes overrepresented in the genes unique to the HepaRG dataset (Figure 7B). The number of unique genes in MCF7 and HepG2 was too small to reliably determine overrepresented processes in these cell lines.

**FIGURE 8:**
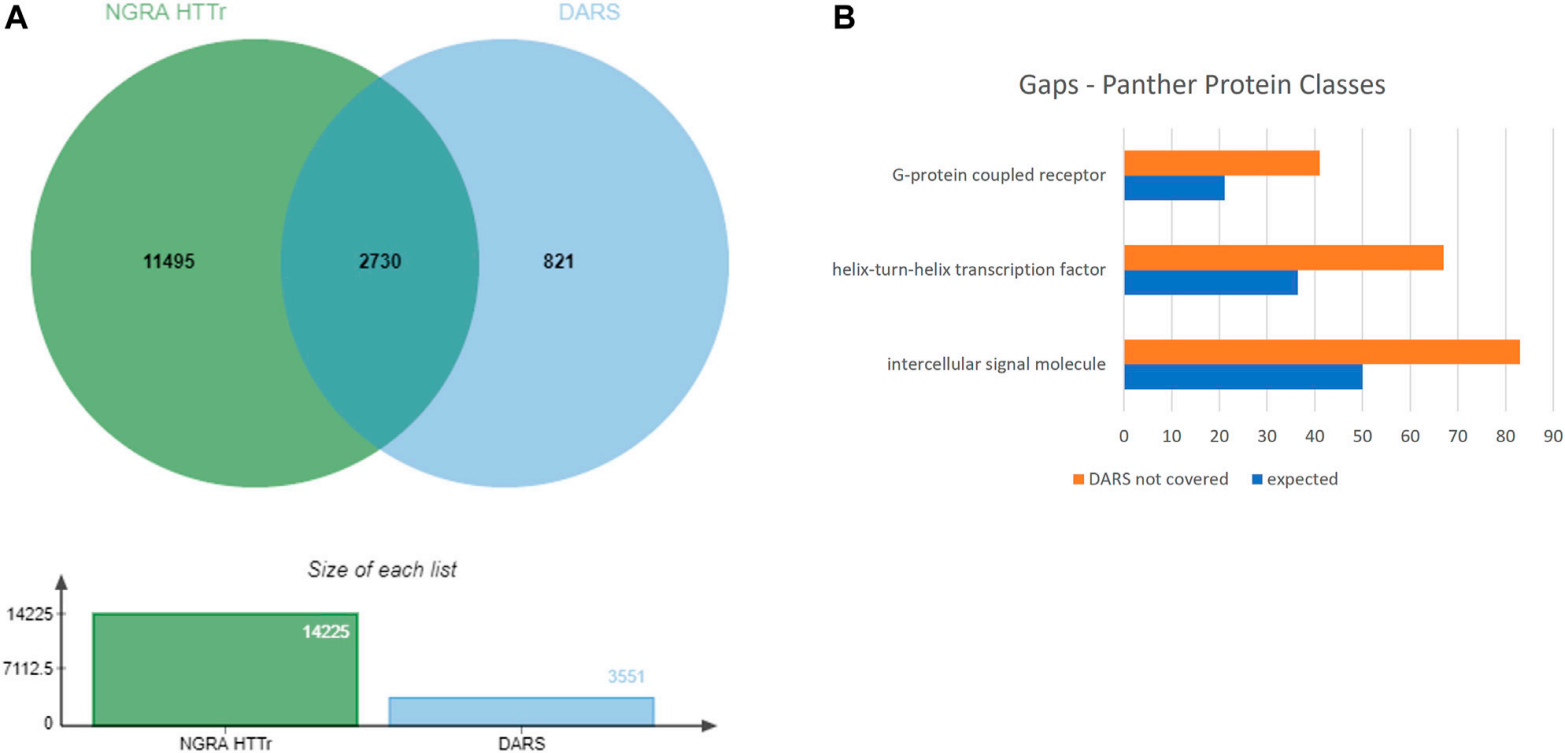
**(A)** Coverage of DARS genes by NGRA HTTr cell lines **(B)** Protein classes represented in the gaps remaining in DARS gene coverage.

The 3,551 DARS gene markers determined from the literature search were compared to the total of 14,225 genes found expressed in the MCF7, HepG2, HepaRG and iPSC cells. 2,730 out of the 3,551 genes were found present in the gene set from the four cell lines (Figure 8A). In particular, transcriptional regulators, protein modifying enzymes, metabolite conversion enzymes, kinase and phosphatase modulators are overrepresented in the intersecting gene list. For the full list of overlapping and unique genes in the two datasets, refer to Supplementary File S8.

#### 5.2.2 Biological Processes

The 474 DARS identified biological processes could be broadly classed into different categories depending on their role in cellular process (Table 2). Direct overlay of these biological processes to pathways or gene expression is difficult due to the fact that multiple signalling cascades may regulate these processes in a redundant or tissue-specific manner.

**TABLE 2:** DARS identified molecular process categorised depending on the cellular function.

Category	Examples
General cellular process	Signalling, DNA methylation, Cell differentiation, Homologous recombination, Cellular metabolic process, etc.
Specific cellular process	Retinol metabolic process, Myelination, Embryonic cleavage, Cytokine secretion, Meiotic cell cycle, etc.
General functional process	Cell migration, Bicellular tight junction assembly, Cell motility, etc.
Specific functional process	Sperm motility, Neuron migration, Axon guidance, Synapse assembly, Macrophage migration, etc.
Specific differentiation	T cell differentiation, Neurogenesis, Hepatocyte differentiation, Erythrocyte differentiation, Cardiocyte differentiation, etc.
Receptor or enzyme activity	1-phosphatidylinositol-3-kinase activity, MAP kinase activity, Interluekin-2 receptor activity, Fibroblast growth factor-mediated receptor activity, Cell adhesion mediator activity, etc.
Signalling pathway	Notch signalling pathway, Nodal signalling pathway, Hippo signalling, Protein kinase B signalling, Wnt signalling pathway, etc.
Cellular stress	Oxidative stress, Heat-shock response, Programmed cell death, Mitochondrial damage, Apoptotic process, etc.
Genotoxicity	Cell cycle checkpoint, Cellular response to DNA damage stimulus, DNA damage checkpoint, DNA repair, Mitotic DNA replication checkpoint, DNA integrity checkpoint, etc.

Several of the general cellular and functional processes would be part of housekeeping or homeostatic mechanisms within a cell and therefore, changes to some of these processes might be indicated through cell survival or cytotoxicity read outs.

The IPP panel, which assesses whether the chemical can interact with several GPCRs, enzymes, ion-channels, transporters, nuclear receptors, and bHLH transcription factors, covers about 13% of the receptor or enzyme activity related biological process key terms. This includes acetylcholine-gated channel activity, NMDA glutamate receptor activity, sodium or potassium channel related activity, serotonin receptor activity, and α_1_/α_2_ and β_1_/β_2_ adrenergic receptor activities.

The signalling pathways’ key markers extracted as part of the DrBP biological processes largely overlap with the DARS genes and for all practical purposes are evaluated together for coverage and gaps.

Formation of the three germ layers and their derivatives are key developmental milestones and is the precursor to specific differentiation to various distinct cell fates (Table 1). This can be mimicked by the differentiation of pluripotent stem cells *in vitro*. The ReproTracker^®^ assay uses human iPSCs differentiated into hepatocytes, cardiomyocytes or neural rosettes, which are representative of cell fates derived from each of the three germ layers; endoderm, mesoderm and ectoderm, measuring the expression of germ line (FOXA2, BMP4 and SOX1) and cell lineage (AFP, MYH6 and PAX6) specific markers genes in a time dependent manner (see Methods). These marker genes are part of the identified DARS markers. However, the differentiation itself covers all signalling networks involved including transcriptomic changes (mRNA expression), posttranslational and epigenetic changes that play a role in the formation of hepatocytes, cardiomyocytes and neural rosettes (Gu et al., 2014; Vasconcellos et al., 2016; Ouyang and Wei, 2021). Assuming that these assays cover most of these three specified differentiation processes, transcriptomic analysis of the ReproTracker^®^ derived tissues would allow a detailed analysis to define the overlap to the whole DARS gene set to get better understanding of the biological coverage of the assay.

The cell stress panel covered key cell stress markers, either as part of normal physiology ensuring survival or development or elimination of damaged or unwanted cells; or activated in response to xenobiotic exposure and stress.

The o/c amino acid ratio, as measured by the devTOX*quick*Predict™ assay, is an indicator of metabolic health of cells and is shown to be a predictive signature of developmental toxicity (Palmer et al., 2013). Ornithine is a non-proteogenic amino acid that plays a role in several biochemical pathways and a decrease in cellular release reflects a perturbation in these pathways. Cystine is used by cells in glutathione production and a decreased uptake from the media is a likely indicator of change in cellular glutathione synthesis and redox balance. Thus, the devTOX*quick*Predict™ assay covered key metabolic signatures of teratogenicity and based on this o/c ratio predicts the potential of a chemical to result in developmental toxicity (Palmer et al., 2013). Additionally, biochemical profiles reflecting MIEs or signalling pathways have been correlated with both its positive and negative predictions (Zurlinden et al., 2020). Zurlinden et al. used a logistic regression strategy considering ToxCast assay specific AC_50_s together with a binary classification outcome model of the o/c ratio to identify sensitive and insensitive pathways associated with the assay predictions (Zurlinden et al., 2020). The annotated records for these MIEs flow largely into either RTK or GPCR signalling. These signalling pathways can be assessed to some extent using HTTr or iPP assays in the DART framework and can further provide mechanistic implications of the o/c ratio. This information can be explored or validated using the existing or additional NAMs.

Based on the training datasets (either publicly available or donated for training purposes) employed, some of the *in silico* tools issue a general alert for developmental toxicity or teratogenicity where as others indicate a specific mode of action, such as those related to the estrogen or androgen receptor signalling mechanisms. DEREK NEXUS includes alerts related to developmental toxicity, teratogenicity and testicular toxicity. The OECD QSAR Toolbox and VEGA both include alerts for developmental toxicants and estrogen receptor (ER)mediated effects. VEGA also includes alerts related to androgen receptor (AR). The OPERA Suite specifically covers estrogen and androgen receptor binding, agonism and antagonism. METEOR covers the prediction of metabolites for compounds in general, which can further be run through each of the above tools for any alert related to teratogenicity or ER and AR modules. MIE Atlas covers a range of alerts, including those related to ER and AR activities. The DART and mode of action related alerts arising from the *in silico* predictions provide a relevant direction of further testing and data generation using either iPP or ReproTracker^®^ or the devTOX*quick*Predict™ assays.

The biological processes that are related to genotoxicity are out of scope of the DART framework as potential for carcinogenicity would be covered as part of an overall safety assessment for a consumer product.

### 5.3 Gaps in Coverage of DARS Markers

Eight-twenty one out of the 3,551 genes were not expressed above the used threshold on expression levels in the four cell lines (see *Methods*). When the missing genes were classified into protein classes using Panther classification system, the over-represented classes were identified as GPCRs, helix-turn-helix (HTH) transcription factors and intercellular signalling molecules (Figure 8B, Supplementary File S9) (Mi et al., 2019). Of the 41 genes in the GPCR class, that lack coverage in the four cell lines, six of them can be evaluated through the iPP panels. Of the 67 HTH transcription factors, 61 are homeodomain transcription factors. The 83 intercellular signalling molecules can be further classed into chemokines, cytokines, growth factors, intercellular signalling molecules, interleukin superfamily members neuropeptides, neurotrophic factor, and peptide hormones.

The specific cellular processes such as cytokine secretion or myelination or androgen biosynthesis cannot be evaluated entirely, given the current set of NAMs. Some of the specific cellular processes such as embryonic cleavage, although specific to developmental biology, may be similarly affected to generic cell division and can be evaluated together with genotoxic effects of chemicals that impact the cell cycle. However, if there are specific spindle poisons or cell division disrupting chemicals to which embryonic cells are more sensitive, those may not be adequately identified using the DART NAMs. Specific functional processes such as sperm motility or axon guidance or lymphocyte migration are also not within the scope of the current NAMs. Although some of cell adhesion related genes can be studied using the HTTr assay, which might add some weight of evidence to cell migratory functions. Differentiation processes such as T cell differentiation or oocyte maturation or Sertoli cell differentiation are also not covered by the ReproTracker^®^ assay or any other assays in the DART framework and will have to be addressed for certain modes of action. Some of the interleukins and cytokines or growth factor receptors or intracellular kinases are not covered by the iPP or CSP assays.

Thus, these gaps that still remain will have to be considered in the context of the mode of action of specific compounds or in a tissue-specific context. While some information in this context could be established from transcriptomic assays (such as differential regulation of genes involved in a biosynthetic pathway or those encoding growth factors), a complete coverage would require either more of the mechanistic data to add to the weight of evidence or data generated in an analogous model system. Similarly, studying specific functional processes may require higher tier models closer to the organoid or micro-physiological conditions. A tiered approach would have to be undertaken to generate additional data.

## 6 Application of the NGRA DART Framework to the Caffeine Case Study

### 6.1 Exposure Estimation

For each exposure scenario, the respective Maternal and Fetal C_max_ was derived. Following the PBK modelling workflow, non-pregnant PBK models were first constructed and verified against available human clinical data. Intravenous infusion data was used for model development by optimising the V_max_ parameter for the CYP1A2 pathway (Supplementary Figure S1A). Then the model was extrapolated into an oral PBK and a dermal PBK model by accounting for their corresponding absorption processes, which were verified by evaluating the model performance against available human clinical studies from oral and dermal exposure routes, respectively, which were not used for model developments (Supplementary Figures S1B,C). In these studies, the model’s predictive performance was evaluated by visual inspection of the time-course curves, as well as calculating C_max_ ratios for observations over simulations. The observed/simulated ratios for C_max_ of all three clinical trials were well within 2-fold limit (calculation not shown), indicating the model predicted values are in good agreement with the respective observed values, therefore both the oral and dermal non-pregnant models were considered reasonable and validated (Jones et al., 2015).

The pregnancy model was further developed based on the non-pregnancy model which accounts for the physiological changes during pregnancy and validated against the plasma kinetics of caffeine after administration of 150 mg single dose in pregnant women. The simulated plasma concentration−time profile captured the observed PK parameters in great agreement (Supplementary Figure S1D). It is worth noting that the changes in maternal CYP1A2 activity have been captured in the software, which is the main enzyme responsible for most of caffeine metabolism. The good match between observed and predicted plasma time profile also indicates that metabolism of caffeine is well predicted too.

The validated pregnant PBK model was then extended to consider the placental and foetal compartments during pregnancy to make predictions on both maternal and foetal concentrations from the two given exposure scenarios. As expected, caffeine exposure was significantly increased as a function of dose across the gestational ages, which is consistent with previous studies reporting a significant prolongation of caffeine elimination half-life in pregnant women.

**FIGURE 9:**
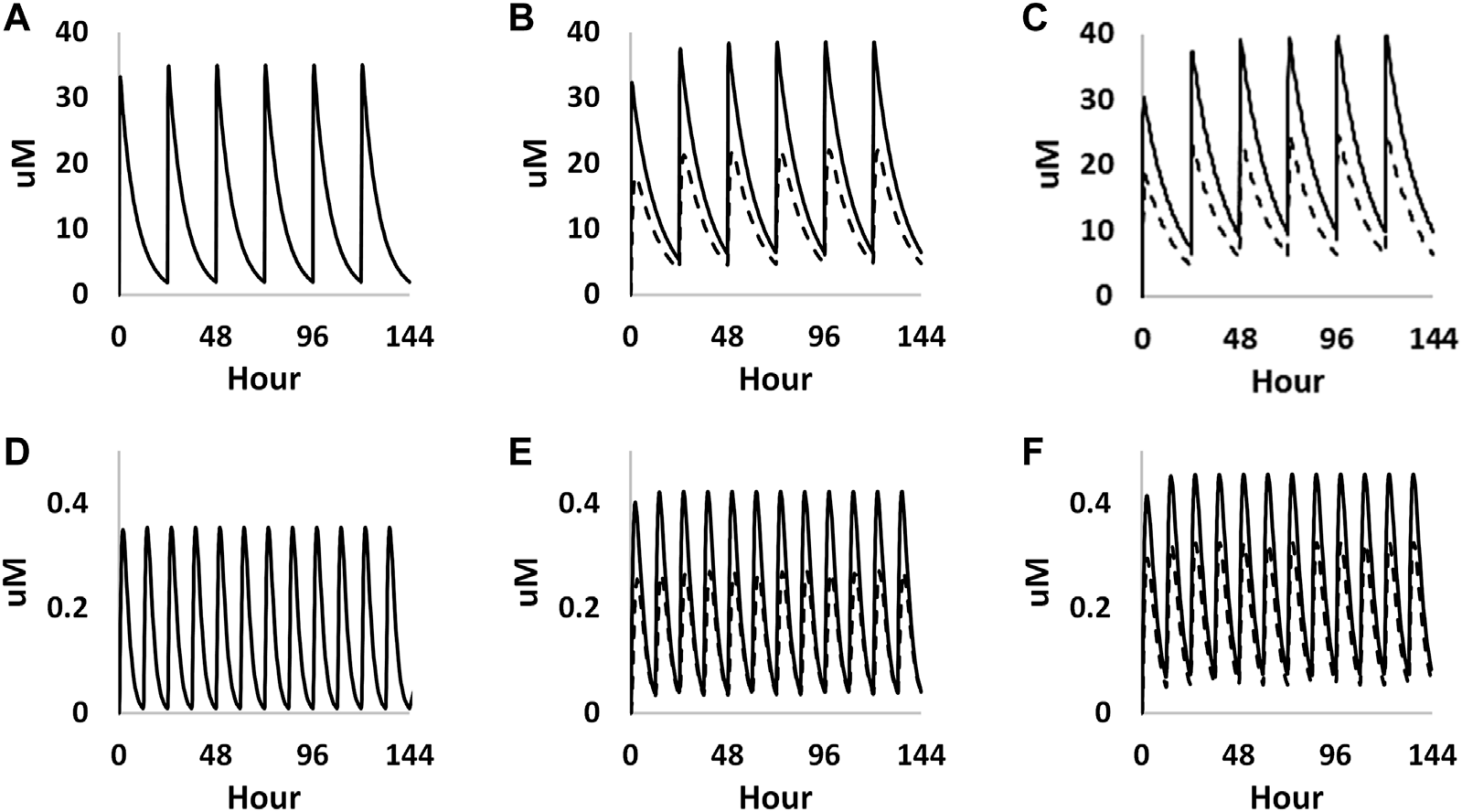
PBK simulations on plasma concentration time profiles of caffeine in both mother (solid curves) and foetus (dashed curves) through different gestational ages. **(A–C)** represent prediction on week 6, week 20 and week 30 from oral exposure, respectively. **(D–F)** represent predations on week 6, week 20 and week 30 from dermal exposure, respectively.

The foetal exposure predictions were not verified due to lack of data on observed foetal concentrations in human clinical studies. The overall ratio between the predicted foetal and maternal (F/M) plasma C_max_ was in the range of 0.57–0.69 for the gestational age greater than 6 weeks (Figure 9 and Table 3). This F/M ratio aligns to F/M ratio after 60 min of perfusion of a term *ex vivo* human placenta (0.79) as well as the ratio reported in the EFSA report (Poulsen et al., 2009; Gundert-Remy 2015).

**TABLE 3:** Summary of the predicted maternal and foetal plasma C_max_ of caffeine at steady state through different gestational ages from both oral and dermal exposure routes.

	Oral: 200 mg/day			Dermal: 0.1% caffeine in body lotion		
	Week 6	Week 20	Week 30	Week 6	Week 20	Week 30
Maternal plasma C_max_ (µM)	34.97	38.51	39.72	0.42	0.42	0.46
Foetal plasma C_max_ (µM)		22.02	25.27		0.27	0.32

### 6.2 Collation of Existing Information

*In silico* tools predicted a wide range of possible toxicity effects based on caffeine’s structure (Supplementary Table S4). DEREK, OECD toolbox and Vega predicted that caffeine has the potential to cause reproductive and developmental toxicity, however it should be noted that these models have been trained on caffeine’s animal experimental data. Other alerts included the potential for protein binding (MIE for skin sensitisation) and genotoxicity. These alerts were outside of the scope of this paper but in a real-life scenario these endpoints would be investigated. There were three positive alerts with high confidence predicted by the MIE ATLAS, namely the AChE (Acetylcholinesterase), ADORA2A (Adenosine receptor A2a) and PDE4A (Phosphodiesterase 4A). Notably, none of tools predicted binding to oestrogen or androgen receptors.

In order to ensure protection of human health, caffeine was experimentally investigated to determine whether it affects these endpoints at consumer relevant exposures using human-relevant tools (IPP, ReproTracker^®^ and devTOXquickPredict™). In addition, CSP and HTTr were performed to increase the biological coverage given that the absence of *in silico* alerts for a given toxicity does not mean absence of effect.

### 6.3 *In Vitro* Biological Activity Characterisation

**FIGURE 10:**
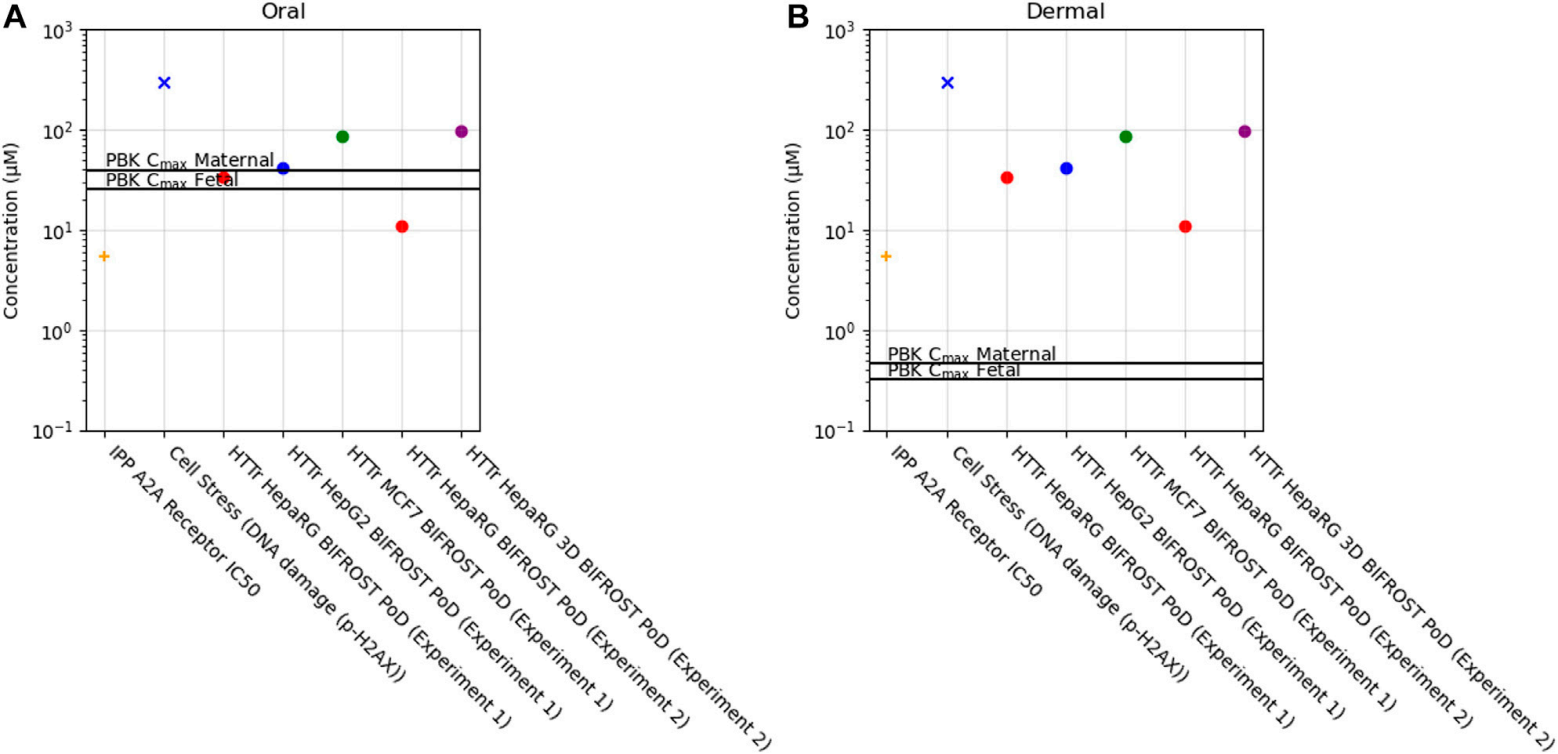
Bioactivity Exposure Ratio of Caffeine for the oral **(A)** and dermal **(B)** exposure scenarios, comparing the IPP, CSP and Httr PoDs with maternal and foetal C_max_ values.

Of the 36 biomarkers in the cell stress panel, caffeine exposure only caused a borderline increase in the biomarker γ-H2AX (phosphorylated H2A histone family member X) (PoD = 304 µM) when tested up to 1,000 µM. Across the different cell models, PoDs derived from HTTr varied between 11 and 96 µM (Figure 10).

In the IPP, the only target hit was the adenosine 2A receptor out of the 60 targets tested (see *Methods* and Supplementary File S4). The follow-up dose-response analysis showed that the IC_50_ (C.I. 95%) for the A2A receptor was 5.3 µM (Supplementary Figure S2). Caffeine is a known non-selective antagonist of adenosine and specifically through the adenosine A2A receptor which is responsible for its psychoactive effect (Jacobson et al., 2020). The other targets predicted by MIE ATLAS were not identified by our panel when caffeine was tested at 10 µM. However, caffeine has also been reported to inhibit multiple phoshpodiasterases (PDE1b, PDE2, PDE3, PDE4, PDE5), GABA_A_ (γ-aminobutyric acid receptor type A), and monoamino oxidase A (MAO-A) at concentrations greater than 10 µM and at not consumer relevant concentrations (Petzer et al., 2013; Pohanka, 2015; Jacobson et al., 2020).

Antagonism of adenosine receptors is a potential cause of concern for cardiac and neuronal tissue embryonic development among other adverse effects in adults (Rivkees et al., 2001; Rivkees and Wendler, 2012; Teerlink et al., 2012; Silva et al., 2013). The ReproTracker^®^ assay included iPSCs that were differentiated towards cardiomyocytes, hepatocytes, and neuronal rosettes in the presence of caffeine. No signs of teratogenicity were observed up to concentrations of 100 µM. Similarly, caffeine did not affect the ornithine/cysteine ratio in the devTOXquickPredict™ assay up to 500 µM.

In summary, the bioactivity data has shown that the most sensitive target was the adenosine 2A receptor.

### 6.4 Determination of Bioactivity Exposure Ratio (BER)

In this case study, we found that a daily consumption of 200 mg caffeine by pregnant women would lead to internal exposure (C_max_) for both the mother and the foetus which was 10–20 fold greater than the most sensitive *in vitro* point of departure (adenosine 2A receptor activity) (Figure 10, Supplementary Table S5). Conversely, for the dermal exposure, the plasma C_max_ was around 10–20 fold lower than the PoD, suggesting that this exposure would not be sufficient to cause *in vivo* bioactivity. For the other biomarkers, BERs were higher but similar trend was observed between exposures, for the oral exposure BER ranged from 0.1 to 12 and for the dermal exposure between 12 and 950 across maternal and foetal predictions (Supplementary Table S5).

Previous studies have shown that a PoD based on the most sensitive pathway or biological response derived from NAMs provides a conservative estimate of the PoD *in vivo* (Thomas et al., 2013; Wetmore et al., 2013; Paul Friedman et al., 2020). In addition, the BER based on the adenosine 2A receptor antagonism would indicate that there is potential DART concern associated with the oral 200 mg/day exposure (BER < 1). However, because bioactivity may not result in an adverse response, the critical safety assessment question is whether this level of activity presents a risk to either the mother or the developing embryo or foetus. Follow-up *in vitro* studies in differentiating cardiomyocytes and neuronal rosettes, which are expected to express adenosine receptors (Headrick et al., 2013), were negative at caffeine exposure up to 100 μM, a concentration that is above the expected level in consumers (∼40–80 µM) and provide reassurance that these levels of activity may not be sufficient to adversely affect cardiac or neuronal development, at least in the periods covered by the assays. This conclusion is supported by the available epidemiology data, which suggest that the most sensitive adverse effect associated with oral caffeine exposure in pregnant women is intra-uterine growth retardation, and not specific malformations of the heart and brain (Gleason et al., 2021).

## 7 Discussion

In this work, we have used the mechanistic knowledge of reproductive and developmental biology to evaluate the coverage and therefore, how fit-for-purpose the NAMs within our safety assessment framework are for DART endpoints. The “*in litero*” approach used to generate the master list of DARS markers combined the systematic categorization of reproduction and development into key stages and morphogenetic events along with use of specific query terms to tap into the existing knowledge base for each of the categories. A pooled master list of 3,551 genes, 474 cellular processes and 338 miRNAs was generated. Just the comparison of these DARS biomarkers genes with the read-outs of the baseline expression of genes in the four cell lines (HepG2, HepaRG, MCF7 and iPSC) and the IPP endpoints used in NAMs that are intended to be part of the DART framework indicated almost 80% coverage of DARS genes.

Of the remaining 821 DARS genes not covered in the basic expression profiles of the four cell lines from NAMs, the predominant classes of proteins are GPCRs, transcription factors with homeodomain transcription factors are the major class, chemokines, cytokines, growth factors, intercellular signalling molecules, interleukin superfamily members neuropeptides, neurotrophic factor, and peptide hormones. Although six of the GPCRs are covered by the iPP panel, the rest are presently not within the direct coverage of the DART framework assays.

Homeobox transcription factors (Hox) play a key role in anterio-posterior and proximo-distal patterning in bilaterian embryos, and their expression is tightly regulated in development (Alexander et al., 2009; Montavon and Soshnikova, 2014). Their expression is closely linked to extension of anterio-posterior body axis and controlled by signalling pathways such a Wnt, Fgf, and RA, as well as the Cdx transcription factors (Dubrulle et al., 2001; van den Akker et al., 2002; Aulehla et al., 2003; Moreno and Kintner, 2004). Similarly, BMP signalling has been shown to pattern the spinal neural tube by regulating Hox transcription factors and the roles of BMP, Shh, Fgf, TSH, IGF1 and TGFβ are well recognized in regulating the thyroid transcription factors, including the Hox TFs, Nkx2-1and Hhex (Timmer et al., 2002; Lopez-Marquez et al., 2021). Since the Hox genes are tightly and combinatorially regulated by growth factor signalling in a tissue specific manner, these upstream activating pathways may provide a better indication of regulation or perturbation of regulation in embryonic development. Indeed, the work by Franzosa et al. has shown that using Bayesian analysis, inferences on MIEs and AOPs can be drawn from differential expression of transcription factors and can be used in an IATA-like approach (Franzosa et al., 2021). Further, transcriptomic analysis of the differentiated cell types from iPSCs as standardized into the ReproTracker^®^ assay may provide germ layer specific or cell type specific niche landscape to probe for expression of these DARS biomarkers.

To assess an impact of chemical exposure on immune system related genes including cytokines, chemokines and interleukins, assays pertaining to its individual components can be considered. Lymphocytic and myeloid cells can be isolated from peripheral blood mononuclear cells (PBMCs) and can be tested to determine toxicity to each of these lineages specifically, upon chemical exposures (Hassan et al., 2007; Pessina and Bonomi, 2007). Other targeted functional assays for specific mechanisms include human lymphocyte activation (HuLA) assay, an antigen recall assay, similar to the *in vivo* T-cell-dependent antibody response (TDAR) where multiple immune cell types are needed to produce responses; multiple cytokines (IL-2, IFN-γ, IL-1β, and IL-8) assay; and the BioMap panel of assays where test systems are constructed with one or more primary cell types from normal human donors stimulated with cytokines or growth factors recapitulate relevant signalling networks that naturally occur in human tissue or disease states and can be explored for specific biomarker readouts (Collinge et al., 2010; Kimura et al., 2018; Singer et al., 2019; Collinge et al., 2020).

Several of the DARS related biological processes are general enough to be indirectly covered either as a cell survival or cytoprotective mechanism or as steady state function to maintain stability or growth. Homologous recombination or endocytosis or DNA methylation would be such an inherent feature of all cells to a large extent. The same rationale can be extended to some of the housekeeping miRNA functionalities as well. However, certain tissue specific mechanisms cannot be completely delineated from the existing NAM models without further validation. A study conducted on human embryonic tissues outlined a high-resolution epigenomic atlas of human craniofacial development (Wilderman et al., 2018). Mapping such functional genomic profiles of embryonic tissues to transcriptomic profiles of cell lines, including iPSCs would further boost the confidence in the mechanistic coverage of NAMs (Haniffa et al., 2021). Multi-generational processes are also challenging to study using generic cell lines with single cell types or those lacking meiotic features. For instance, studying parental exposure to compounds that may have an effect on embryo-foetal development in subsequent generation, such as sperm mediated developmental toxicity by means of epigenetic programming, including sperm DNA and histone modifications and non-coding RNAs in spermatozoa would require additional cell models with adequate mechanistic similarity or coverage (Bonde et al., 2016; Marcho et al., 2020). Furthermore, developing some of the tissue specific models have more inherent challenges than others and need additional considerations. For example, using the ReproTracker^®^ set up to differentiate into additional cell fates such as osteoblast or other stromal lineages would be relatively straight forward compared to building an *in vitro* set up recapitulating the human testis (Oliver and Stukenborg, 2020). Placental toxicity is another key cell type specific aspect of DART (Gingrich et al., 2020). However, the *ex vivo* or other advanced models of human placenta are still in their early stages of development (Hougaard, 2021).

Spatio-temporal and tissue-specific regulation is another critical aspect of developmental systems. This is key to the action of long-range signals such as growth factors and hormones and is well exemplified in regulation of both morphological (e.g., somitogenesis) and molecular processes (e.g., epigenetic modifications) (Maroto et al., 2012; He et al., 2020). While this work assesses the coverage of the key signalling and cellular machinery in reproduction and development, and the NAMs discussed do provide an opportunity to test time-dependent effects to certain extent, the entirety of spatio-temporal control as in a human embryo may not exist within a single NAM assay. The conservative outputs or PoDs from the NAMs described can be used directly in assessing risk, as a protective approach. However, these outputs cannot be directly related to the resulting cellular or morphological changes that may take place in a human embryo. Therefore, if a safety decision cannot be reached using this protective approach further targeted investigations or refinements will be needed. These may include the use of higher-tier approaches, such as hiPSC derived embryos combined with single cell omics techniques (Shahbazi et al., 2019; Zheng et al., 2019; Xiang et al., 2020; Sozen et al., 2021), bespoke microphysiological systems (MPS) and conceptual or agent-based or dynamic models (Kleinstreuer et al., 2013; Villeneuve et al., 2014; Capone et al., 2018; Thomas et al., 2019; Richards et al., 2020; Kim et al., 2021; Wiedenmann et al., 2021), to make a more explicit link between the cellular changes observed and an adverse outcome (a quantitative AOP). Such a bespoke approach will require a significant investment that may not be practical for day-to-day decision making.

MiRNAs have emerged over the last few years as important modulators of signalling networks in foetal development by regulating developmental transitions, lineage specification, and securing cellular identities (Ebert and Sharp, 2012; Ivey and Srivastava, 2015; Prodromidou and Matsas, 2019). Tissue specific microRNAs are found to be dynamically regulated in a cell lineage and developmental stage-specific manner (Vasconcellos et al., 2016; Ouyang and Wei, 2021). These dynamic changes can also be observed *in vitro*. Kim et al. showed specific temporal expression of miR-1, -30d, -133a, -143, -145, -378a, -499a during differentiation of human embryonic stem cells into a cardiac lineage (Kim et al., 2017), some of which were also identified in our literature search as important regulators for heart development (Supplementary File S7). So, while bioinformatic evaluation of the miRNA DARS markers was out of scope for this work we believe that miRNA expression is at least partially covered in all cell lines used as well as in the iPSC and the differentiating cells of the NAMs discussed for the DART framework. However, using miRNA analysis complementarily to mRNA analysis could bring further advantages for *in vitro* toxicity testing in future. Analogous to transcription factors, one miRNA can regulate several mRNAs influencing different functional processes at once. MIR-21 (second highest score in the literature extraction, Supplementary File S7) is found to affect the expression levels of many different genes from different pathways and molecular events regulating epithelial-to-mesenchymal transition, a biological process essential for organ development (Huntley et al., 2018). While knockout of single miRNAs often produces subtle phenotypes under homeostatic conditions overexpression or misexpression of a single miRNA can promote remarkable alterations in cell differentiation and can even induce differentiation of multi- or pluripotent cells *in vitro* (Shenoy and Blelloch, 2014; Channakkar et al., 2020; Jaafarpour et al., 2020). Dose-dependent perturbations of miRNA upon chemical exposures (Smirnova et al., 2014) and its link to the development of diseases for humans *in vivo* have been reported (Schraml et al., 2017; Wallace et al., 2020). Especially the fact that circulating miRNAs in the maternal blood can be used as biomarkers for pregnancy complications and for detecting foetal abnormalities (Yang et al., 2020) make them important clinical biomarkers and could also help identify new mode of actions for developmental toxicity.

A workshop reviewing the application of the nine ICCR principles to NGRA case studies and how can these principles build confidence in safety decision making, using NAMs, identified the evaluation of the biological coverage as one of the seven areas that would help to make NGRA useful for cosmetic ingredients (Dent et al., 2021). Either using a multitude of cell and tissue types or focussing on key pathways and mechanisms of concern irrespective of the origin of the cell type are two approaches that can be used alternatively or in combination to address safety concerns. The current approach of generating a reference list of DARS biomarkers enables such a robust evaluation of DART related biological coverage. Overall, this evaluation found the NAMs to broadly cover the key signalling processes and mechanisms underlying the apical endpoints in traditional DART studies, such as those specified within the OECD guidelines and undertaken for regulatory purposes, thereby enabling their assessment in a tiered manner to be protective of human health. This approach also provides the opportunity to identify gaps and address them through the above-mentioned propositions of either additional AOP-based or cell type specific analysis. Furthermore, the individual list of DARS markers available for each of the stages can be used as either foundational or supporting knowledge for DART endpoint-specific AOPs, thereby enabling a refinement in NGRA.

One of the objectives of this paper was to illustrate how the NAMs within the Developmental and Reproductive Safety Framework can be protective of DART effects spanning the key stages in reproduction and development. We followed this framework for two hypothetical exposure scenarios, applying the PBK model approaches to estimate caffeine’s internal maternal and foetal exposure, combined with the biological activity characterisation using the NAMS evaluated in the biological coverage section. Caffeine effects during pregnancy have been a topic of debate for many years, but safety agencies around the world agree that a daily consumption of 200 mg is unlikely to lead to significant adverse reproductive and developmental effects (USDA 2015; EFSA, 2015; FDA, 2018; ACOG, 2020; Gleason et al., 2021; James, 2021; NHS, 2020). Here we found that an oral dose below 20 mg/day and a dermal exposure with 0.1% caffeine would result in a maternal BER higher than one implying that this exposure would result in no *in vivo* biological activity. It should be noted that this exposure does not take into account any uncertainties associated with e.g. inter-individual variability in the pharmacodynamics and pharmacokinetics (Nehlig, 2018). However, given the existing knowledge of caffeine, this level would clearly be protective of human health. This case study focused on the risk assessment of caffeine without considering the potential exposure and toxicity of its metabolites. We have, however, incorporated the reduced caffeine metabolism observed during pregnancy (decrease in CYP1A2 activity) which leads to higher caffeine plasma concentrations (Tracy et al., 2005). As a further refinement, the risk assessment should consider the biologically active metabolites, paraxanthine, theophylline, and theobromine (Institute of Medicine et al., 2001; Orru et al., 2013).

The caffeine case study is a practical example that demonstrates how to integrate relevant NAMs for safety decisions without generating new animal data and in this instance, the tools provided a conservative estimate of risk. However, teratogens and reproductive toxicants are known to act through multiple mechanisms of toxicity (Mattison and Thomford, 1989; van Gelder et al., 2014) which means that our NAM toolbox might not provide adequate biological coverage to all chemicals. Therefore, to build confidence that NAMs can be protective of DART effects for a wide range of chemicals, we need to generate larger datasets for multiple types of chemistries, toxicity modes of action and exposure scenarios. Work is ongoing to generate the NAM toolbox data for a substantial number of known human teratogens and non-teratogens with known human exposure. This approach can be used not only to evaluate how protective the toolbox is but also to identify potential areas of refinement (e.g., application of higher tier tools triggered by molecular signatures or specific targets) and to develop new tools (e.g., to address gaps in the biological coverage). Formulating more and more DART specific case studies and using the NAMs data to guide the evaluation of novel chemicals will help realise the ambition of NGRA and non-animal alternates for safety decision making.

Lastly, we are yet to have a unified database as a resource that can share the available information on epidemiological and environmental aspects or clinical biomarkers for the large number of industrial chemicals and pharmaceutical compounds (Hougaard, 2021). Such a database would be immensely valuable in validating and benchmarking NAMs outputs with the gold standard human evidence. A clinical study of prenatal exome sequencing analysis, after excluding for extra chromosomes or large deletions or duplications of chromosomes, identified a list of genes associated with foetal structural anomalies (Lord et al., 2019). Roughly 50% of these genes overlapped with the DARS markers which was a good validation of the DARS list as not all alleles associated with prenatal defects resulted in a loss of function. Indeed, a collaboration between toxicologists, epidemiologists and clinicians studying birth defects and reproductive disorders would strengthen and expedite the validation and application of NAMs towards safety of human health. A workshop or a similar platform to enable such cross-talks would be very valuable in the future.

## Data Availability

The original contributions presented in the study are included in the article/Supplementary Material, further inquiries can be directed to the corresponding author.
