# Supplementary material for: Beyond AOPs: A Mechanistic Evaluation of NAMs in DART Testing
Source: Front Toxicol. 2022 Mar 7;4:838466. doi: 10.3389/ftox.2022.838466 (PMC8915803; doi:10.3389/ftox.2022.838466)
Supplement: Supplementary file 9 [file DataSheet1.DOCX]

Contents

[Supplementary Figures 2](#_Toc89243678)

[**Figure S1** Comparison on plasma concentration time profiles of caffeine between PBK simulations and observed human clinical PK data 2](#_Toc89243679)

[**Figure S2**. Dose response curve used to generate IC50. 3](#_Toc89243681)

[Supplementary Tables 4](#_Toc89243682)

[**Table S1.** Physiochemical and ADME parameters of caffeine used as input in the PBK models 4](#_Toc89243683)

[**Table S2.** Use scenarios of consumer exposure to body lotion 5](#_Toc89243684)

[**Table S3.** In Silico Tools and corresponding DART-relevant models 5](#_Toc89243685)

[**Table S4**. Summary of *in silico* predictions for caffeine 6](#_Toc89243686)

[**Table S5.** Bioactivity exposure ratio (BER) obtained from each technology for both maternal and foetal exposure. 7](#_Toc89243687)

# Supplementary Figures

## **Figure S1** Comparison on plasma concentration time profiles of caffeine between PBK simulations (solid/dashed curves) and observed human clinical PK data (solid/open dots), A: 30 min intravenous infusion of 4.85 mg/kg caffeine (Vmax was optimized fitting against this data for non-pregnant model development); B-1: oral administration of 4.94 mg/kg caffeine for non-pregnant oral model verification; B-2: oral administration of 70 mg and 200 mg of caffeine for non-pregnant oral model verification; C: topical application of 1.25 mg caffeine on 25cm2 chest, for non-pregnant dermal model verification; D: oral administration of 150 mg caffeine in a pregnant woman for pregnant model verification

## ****

## **Figure S2**. Dose response curve used to generate IC50 = 6.4 uM for binding of caffeine to Adenosine receptor A2a. 95% C.I. was determined to be between 5.3 uM and 7.6 uM.


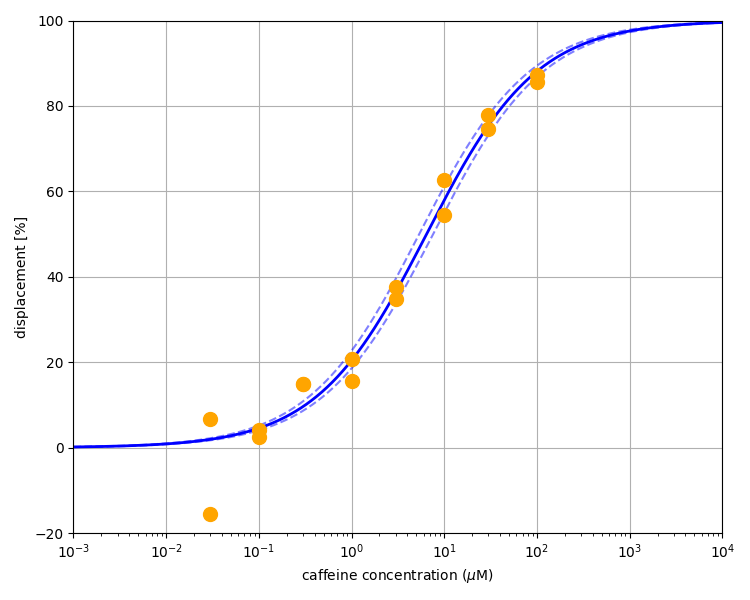


# Supplementary Tables

## **Table S1.** Physiochemical and ADME parameters of caffeine used as input in the PBK models

| **Parameter** | **Value** | **Source** |
| --- | --- | --- |
| LogP | -0.07 | {Hansch, 1995 #217} |
| Solubility (mg/mL) | 0.988 | Experimental value, 20 °C in phosphate buffer {Moxon, 2020 #201} |
| Fup | 0.68 | {Lelo, 1986 #218} |
| Rbp | 0.8 | Experimental value {Moxon, 2020 #201} |
| CYP 1a2 V_max_ (mg/s/mg-enz) | 0.0015 | Optimized against i.v. infusion PK data |
| CYP 1a2 Km (mmol/L) | 0.19 | {Ha, 1996 #216} |
| Renal clearance (L/h) | 0.00246 | {Birkett, 1991 #215} |
| Effective permeability for intestinal absorption (cm/s * 10^4) | 6.9 | Calculated based on Papp=4.63-5.35 10-5 cm/s using COVca method,{Smetanova, 2009 #214} |
| diffusifity in stratum corneum (cm2/s) | 1.4E-09 | {Moxon, 2020 #201} |
| stratum corneum/water partition coefficient | 1.96 |  |
| diffusifity in viable epidermis (cm2/s) | 6.7E-09 |  |
| viable epidermis/water partition coefficient | 1.37 |  |
| diffusifity in dermis (cm2/s) | 3.1E-06 |  |
| dermis/water partition coefficient | 1.04 |  |
| Apparent permeability coefficient for placental transfer (cm/s * 10^4) | 3.2 | Determined at 60 min in BeWo b30 transwell experiment {Poulsen, 2009 #219} |

## **Table S2.** Use scenarios of consumer exposure to body lotion

| **Product types** | **Body lotion** |
| --- | --- |
| Amount of product used per day (g/day) using 90th percentile | 7.82 {Hall, 2007 #2} |
| Frequency of use | 2 time/day ^c^ {Bernauer, 2018 #71} |
| Amount of product in contact with skin per occasion (mg) | 3910 |
| Ingredient inclusion level | 0.1% |
| Application site | Whole body (excluding head) |
| Skin surface area (cm^2^) | 15670 ^d^ {Bernauer, 2018 #71} |
| Leave on or rinse off | Leave on |
| Exposure duration per occasion | 12 hours |
| For rinse off product, retention factor of finished product on skin ^e^ | n.a. |
| Amount of ingredient in contact with skin per occasion (mg) | 3.91 |
| Local dermal exposure per occasion (µg/cm^2^) | 0.25 |

## **Table S3.** In Silico Tools and corresponding DART-relevant models

| **In Silico Tool** | **Profiler** | **DART-relevant model** |
| --- | --- | --- |
| DEREK NEXUS (v6.1.1) (Lhasa limited): | Reproductive toxicity | Developmental toxicity |
|  |  | Teratogenicity |
|  |  | Testicular toxicity |
|  |  |  |
| Meteor Nexus (v3.1.0) (Lhasa limited) | Metabolites |  |
| OECD QSAR Toolbox (v 4.5 2021) [ref]: | Mechanistic profilers | Estrogen receptor binders |
|  | Endpoint Profilers | DART Scheme |
|  |  | rtER Expert System – USEPA |
| VEGA (v1.15) [ref]: | Reproductive toxicity | Developmental / Reproductive Toxicity library (PG) |
|  |  | Estrogen receptor relative binding affinity model (IRFMN) |
|  |  | Estrogen receptor-mediated effect (IRFMN / CERAPP) |
|  |  | Androgen receptor-mediated effect (IRFMN / COMPARA) |
| OPERA (v2.7) [ref]: | ER activity (CERAPP) | Agonist, Antagonist, Binding |
|  | AR activity (CoMPARA) | Agonist, Antagonist, Binding |
| MIE Atlas (Unilever) | ER activity | Binding |
|  | AR activity | Binding |

## **Table S4**. Summary of *in silico* predictions for caffeine

| **Tool/model** | **Alerts/Predictions** |
| --- | --- |
| DEREK | Positive alerts for Chromosome damage in vitro, ocular toxicity and teratogenicity. (Plausible and probable) |
| METEOR | 28 metabolites were predicted, 14 of which were unique. Most common biotransformation = Oxidative N-Demethylation |
| DEREK - Metabolites | All 14 unique metabolites reported positive alerts in DEREK. The alerts reported included teratogenicity, ocular toxicity and chromosome damage in vitro. There were no new alerts compared to parent caffeine |
| OECD QSAR Toolbox | Positive alerts were reported by the following profilers:   - - Blood brain barrier: poor permeability   - DNA binding by OASIS: Alert: SN1 iminium ion formation   - Oral absorption: Highly absorbed   - Protein binding by OECD: Alert: Acylation   - Skin permeability: High permeability   - Cramer: High (Class III)   - DART Scheme: Known precedent reproductive and developmental toxic potential – purine and pyrimidine-like derivatives (7b)   - In vivo mutagenicity (micronucleus): H-acceptor-path3-H-acceptor   - Keratinocyte gene expression: High gene expression N-Acrylamides   - Protein binding alert for chromosomal aberration by OASIS: Alert AN2 |
| VEGA | The following positive results were reported:   - - Predicted Oral Carcinogenicity SF [1/(mg/kg-day)]: 0.9142   - Predicted Inhalation Carcinogenicity SF [1/(mg/kg-day)]: 0.3071   - Developmental toxicant (no data on reproductive toxicity) (EXPERIMENTAL value)   - Skin sensitisation: Sensitizer (low reliability)   - Hepatotoxicity:  Toxic (EXPERIMENTAL value)   - Adipose tissue blood model:  -0.638 (EXPERIMENTAL value)   - Total body elimination half-life: 0.69 (EXPERIMENTAL value)   - In vitro micronucleus activity: Active (EXPERIMENTAL value)   - Predicted NOAEL [mg/kg]: 1.76 |
| OPERA | Results for ER and AR models (agonist, antagonist and binding activity) were all negative.  CATMoS predicted LC50 = 244.00 |
| MIEs Atlas | There were 3 positive alerts (with high confidence):   - - AChE (Acetylcholinesterase)   - ADORA2A (Adenosine receptor A2a)   - PDE4A (Phosphodiesterase 4A) |

##

## **Table S5.** Bioactivity exposure ratio (BER) obtained from each technology for both maternal and foetal exposure.

| **Technology** | **Point of departure (PoD) (µM)** | **BER oral exposure (200 mg caffeine)** | | **BER dermal exposure (0.1% in body lotion)** | |
| --- | --- | --- | --- | --- | --- |
|  |  | **Maternal** | **Foetal** | **Maternal** | **Foetal** |
| IPP A2A Receptor IC50 | 5.3 | 0.1 | 0.2 | 12 | 17 |
| Cell Stress (DNA damage (p-H2AX)) | 304 | 8 | 12 | 661 | 950 |
| HTTr HepaRG BIFROST PoD (Experiment 1) | 34 | 0.9 | 1 | 74 | 106 |
| HTTr HepG2 BIFROST PoD (Experiment 1) | 42 | 1 | 2 | 91 | 131 |
| HTTr MCF7 BIFROST PoD (Experiment 1) | 85 | 2 | 3 | 185 | 266 |
| HTTr HepaRG BIFROST PoD (Experiment 2) | 11 | 0.3 | 0.4 | 24 | 34 |
| HTTr HepaRG 3D BIFROST PoD (Experiment 2) | 96 | 2 | 4 | 209 | 300 |
